# Dietary niches of terrestrial cercopithecines from the Plio-Pleistocene Shungura Formation, Ethiopia: evidence from Dental Microwear Texture Analysis

**DOI:** 10.1038/s41598-018-32092-z

**Published:** 2018-09-19

**Authors:** Florian Martin, Chris-Alexander Plastiras, Gildas Merceron, Antoine Souron, Jean-Renaud Boisserie

**Affiliations:** 1 Laboratory Paleontology Evolution Paleoecosystems Paleoprimatology (PALEVOPRIM) - UMR CNRS-INEE/University of Poitiers, 86073 POITIERS CEDEX 9, France; 2 School of Geology, Aristotle University of Thessaloniki, 52124 THESSALONIKI, Greece; 3 De la Préhistoire à l’Actuel: Culture, Environnement et Anthropologie (PACEA) – UMR 5199 CNRS/Université de Bordeaux/Ministère de la Culture et de la Communication, 33615 PESSAC CEDEX, France; 4 Centre Français des Etudes Ethiopiennes (CFEE) – USR 3137 CNRS/Ministère de l’Europe et des Affaires Etrangères, Ambassade de France en Ethiopie, ADDIS ABABA, Ethiopia

## Abstract

This study aims to explore the feeding ecology of two terrestrial papionins, *Papio* and *Theropithecus* from the Shungura Formation in Ethiopia, the most complete stratigraphic and paleontological record of the African Plio-Pleistocene. Two aspects were evaluated using Dental Microwear Texture Analysis: differences in diet between the extinct genera and their extant relatives, and any potential dietary fluctuations over time. Amongst more than 2,500 cercopithecid dental remains, 154 *Theropithecus* molars and 60 *Papio* molars were considered. Thirty-nine extant wild baboons and 20 wild geladas were also considered. The results show that diets of extinct monkeys from Member G already differed between genera as it is the case for their extant representatives. The shearing facets on the *Theropithecus* molars display significant variations in microwear textures, suggesting several dietary shifts over time. Two events point to higher intakes of herbaceous monocots (tougher than dicots foliages), at about 2.91 Ma (between members B and C) and at 2.32 Ma (between members E and F). These two events are separated by an inverse trend at about 2.53 Ma (between members C and D). Some of these variations, such as between members E and F are supported by the enamel carbon isotopic composition of herbivorous mammals and with paleovegetation evidence.

## Introduction

**Figure 1:**
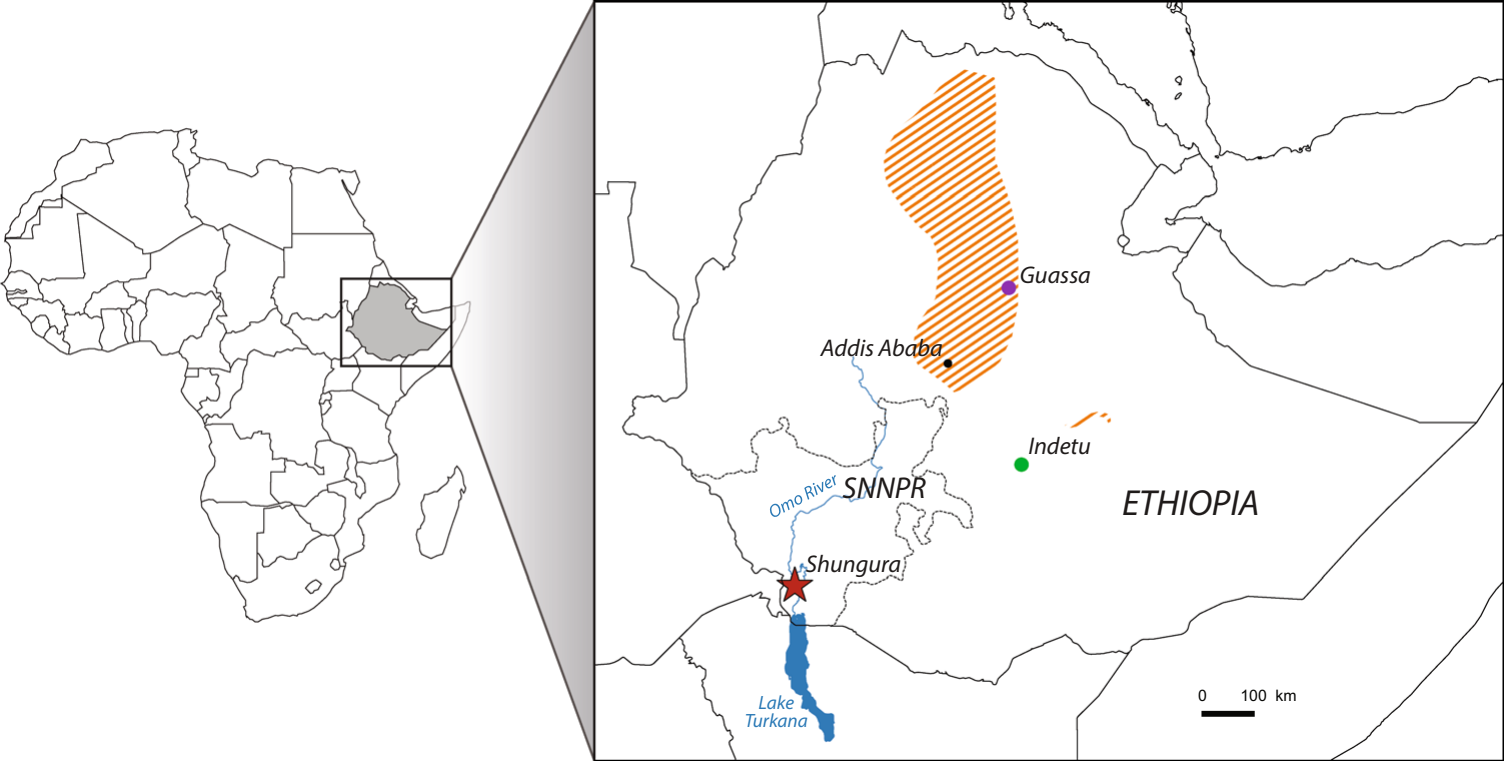
Geographical context of the study. Localization of the Plio-Pleistocene deposits of the Shungura Formation; geographical extent of the subsistence area of the extant gelada (in striped orange; IUCN 2017); and localization of gelada populations from Guassa and Indetu, respectively studied by Fashing *et al*.^35^ and Abu *et al*.^77^. See also Welch *et al*.^78^ for an updated and more detailed geographical distribution of the gelada.

*Papio* and *Theropithecus* are terrestrial cercopithecids whose extinct relatives were more diverse and widespread. For example, the modern gelada (*Theropithecus gelada*) is a refugee species in the Ethiopian High Plateau, with a restricted geographical range and likely a reduced set of habitats compared to its extinct relatives (Fig. 1). The present work aims to investigate the ecological segregation between these two primate genera found in eastern African Plio-Pleistocene sites. The second goal is to track changes in the feeding habits of these cercopithecids that reflect variations in food resources over time, in the context of environmental changes during the Plio-Pleistocene at a regional scale. To do so, dental microwear textures of specimens of *Papio* and *Theropithecus* from the Shungura Formation were analyzed. Shungura is a geological formation situated within the Lower Omo Valley, on the northern part of the Turkana Basin in southwestern Ethiopia (Fig. 1). Its sedimentary deposits have yielded major Plio-Pleistocene paleontological and archeological records, including numerous hominin and lithic remains. It is subdivided into 12 geological members, from the lowermost to the uppermost: Basal, A to H, and J to L. The main volcanic tuffs of the formation, forming the base of each member, allow for long distance stratigraphic correlations. These and other ash layers have been dated by radiochronological methods, including ^40^K/^40^Ar and ^40^Ar/^39^Ar, and by magnetostratigraphy. These methods and extrapolations from their results are indicative of a time interval of 3.6 Ma to 1.05 Ma for the deposition of the formation. This in turn led to a particularly accurate chronostratigraphic framework for testing evolutionary hypotheses. Unlike all other African Plio-Pleistocene sites, this formation displays a good chronological continuity of its sedimentary deposits, notably between 3 Ma and 2 Ma, documenting a critical period in hominin evolution marked by the transition between *Australopithecus* and *Homo* and by the emergence of *Paranthropus*.

The inter-specific and temporal variations in the dental microwear textures of these terrestrial monkeys were compared with enamel stable carbon isotopic data and paleovegetation evidence. This provides insights into the available vegetation in the cercopithecid habitats along the ancestral Omo River and therefore allows for identifying environmental trends.

**Figure 2:**
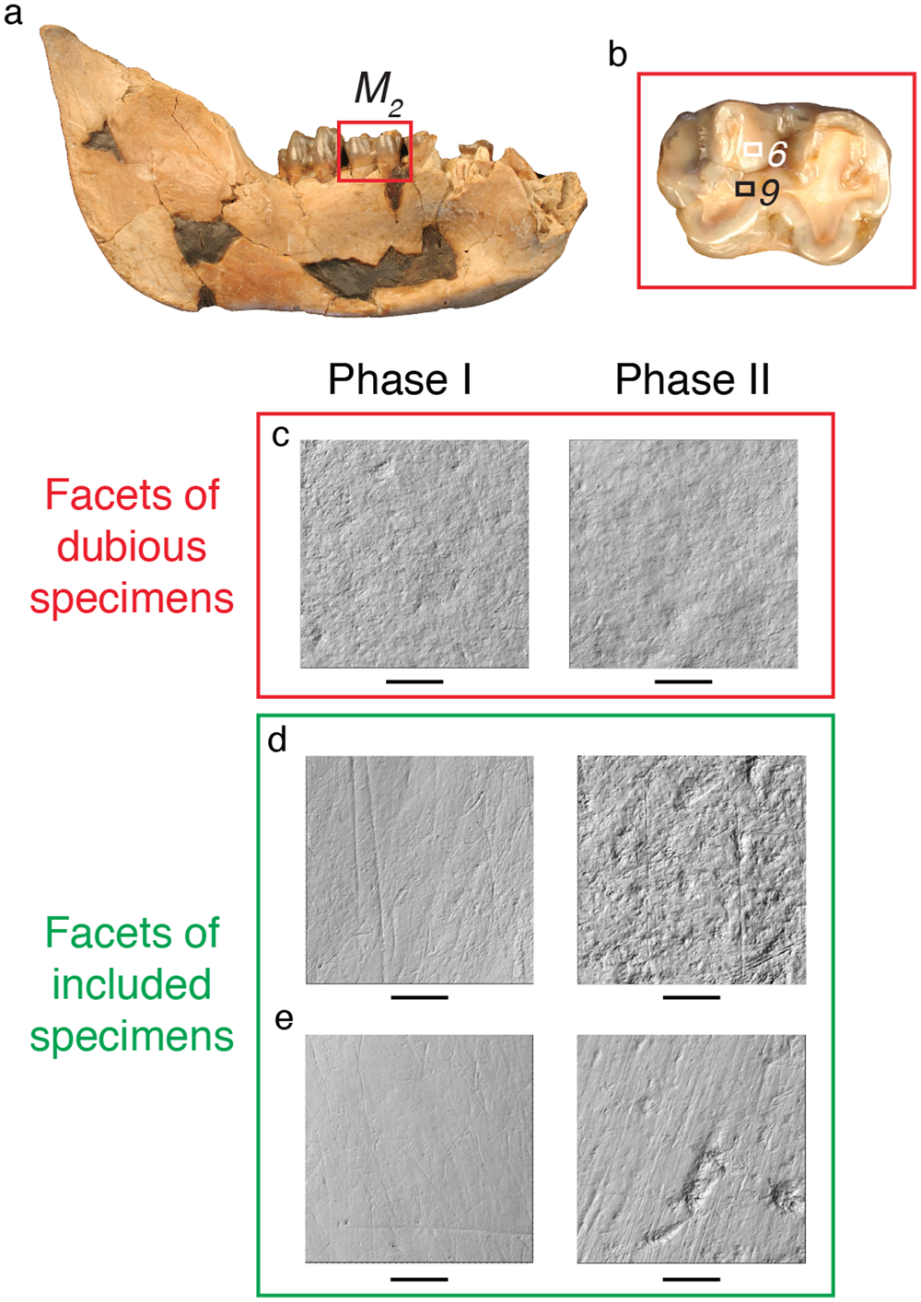
From the fossil remain to the dental microwear textures. (**a**) Fossil mandible (L 199-5) attributed to *Theropithecus brumpti* and recovered in Member C of the Shungura Formation. (**b**) Right lower second molar from the mandible in occlusal view with the localization of the two scanned facets. (**c**) Photosimulations of the two types of facets on the lower third molar (respectively facet 8 and facet 12) of a dubious specimen, OMO 18-1968-2238, that were not included in the study due to potential postmortem alteration. Pairs of photosimulations showing microwear textures differing between the two types of facets and that were included in the analyses; (**d**) OMO 28-1968-1273, lower third molar with facets 5 and 11; (**e**) OMO 18-1969-510, upper second molar with facets 3 and 9. Scale bars represent 50 µm.

Most primate remains found in the Shungura Formation belong to *Papio* and *Theropithecus*. The latter taxon has a unique dietary specialization among extant primates: it forages predominantly on the herbaceous layer and mostly on monocotyledons. Today, *Papio* is represented by several species throughout Africa. Up to six species have been recognized by now, but the phylogeny and systematics have been debated for decades^1–4^. These extant monkeys are terrestrial and have a wide spectrum of feeding habits^5–11^, which include the aerial and subterranean parts of herbaceous vegetation, and also flowers, leaves, fruits and seeds from the shrub to the arboreal layers, as well as animal matter (see references and Fig. 2, p. 556 in Scott *et al*.^12^).

As fossil *Papio* and *Theropithecus* present the same different dental morphologies met between their extant relatives, evidence that diets differed between extinct taxa was expected. Based on enamel stable carbon isotopic composition, Lee-Thorp *et al*.^13^ discovered that the Swartkrans baboons fed on C_3_ vegetation, whereas the sympatric species of *Theropithecus* mostly consumed C_4_ vegetation. Taking into account the differences in dental morphology, this result was to be anticipated. Other studies have also examined stable carbon isotopes in the enamel of fossil *Theropithecus*^14–19^. Although this dietary proxy provides information on the proportions of plants using C_3_ and C_4_ photosynthetic pathways in the diet, it cannot distinguish the type of organs consumed. Dental Microwear Texture Analysis (DMTA) offers a complementary source of data that gives information on the mechanical properties of the eaten organs for more complete inferences of the dietary habits of these fossil representatives. Indeed, each organ has its own mechanical properties and inner composition^20–22^. We expect mature foliages of silica-bearing herbaceous monocots to generate less complex and more anisotropic microwear textures on primate teeth than the consumption of rhizomes, bulbs or small seeds from ear^12^. There is no doubt that underground storage organs are ingested with grit that contribute to tooth wear. We also expect primates foraging on a wide spectrum of food types (with different mechanical properties) to have more variable dental microwear textures than a monkey focusing for instance only on foliages. Such variations has been found on different modern ungulates with different feedings habits but also on captive animals fed with different fodders^23–27^.

## Materials and Methods

### Materials

All the fossil specimens considered in this study were collected by the International Omo Research Expedition (IORE, 1967–1976) and the Omo Group Research Expedition (OGRE, since 2006) in the Shungura Formation, Lower Omo Valley, Ethiopia. They were all gathered between Member B and the lower part of Member G (stratigraphic units 1–13), covering a time span of 3.44 Ma to 2.05 Ma (see Appendices 1 and 2).

The sediments constituting these stratigraphic entities were mostly deposited in a fluviatile context, with the recurrence of a sequence of sands and sandstones at the bottom, silts, and clays/clayey silts at the top. These were respectively interpreted as channel deposits, levee deposits, and flood plain deposits^28^. These sediments include volcanic tuff deposits, for which the sedimentary facies strongly suggest underwater deposition in a fluviatile context.

The specimens studied here were essentially found in the sandy horizons, with the major exception of the specimens from OMO 33 that came from a sandy-tuffitic deposit, again in a fluviatile context. The Shungura Formation also includes a large number of archeological occurrences in Member F and the lower part of Member G^29,30^, almost exclusively containing lithics made from quartz^31^. However, most of these elements occur in silts or clays. They do not co-occur with fossil concentrations, and are not associated with significant faunal assemblages. The material studied here was not discovered in such archeological contexts, but in natural assemblages. The radiometric ^40^Ar/^39^Ar dates from McDougall and Brown^32^ and McDougall *et al*.^33^ are used here for dating the boundaries between geological members.

More than 2,500 dental remains of Shungura cercopithecids attributed with certainty to the genus *Theropithecus* and 173 attributed with certainty to the genus *Papio* are stored at the Authority for Research and Conservation of Cultural Heritage in Addis Ababa. Among all of these specimens, 297 dental remains of *Theropithecus* and 110 of *Papio* were selected for analysis because their molars display apparent functional dental facets without post-mortem damage. Initially, observations were made with the naked eye or with a 10 × handheld lens. When non-occlusal and occlusal surfaces showed similar alterations, this was a hint of post mortem damage that allowed the exclusion of the specimens before molding. The next step happened after molding and scanning (Fig. 2). Dental microwear textures between shearing and crushing facets are expected to differ from each other, as the two facets contribute to different phases during mastication. So contrasted dental microwear textures were indeed a clear hint that the dietary signal was preserved. When the two facets showed identical pitted enamel surfaces without any scratches, the specimen was rejected from the analysis.

Then, among those, DMTA has been performed on 154 *Theropithecus* individuals and 60 *Papio* individuals (see Appendices 1 and 2). Post-mortem damaged molars are identifiable using the following characteristics: homogeneous rough textures with similar small pits and an absence of apparent scratches on both Phase I and Phase II dental facets. Furthermore, scratches and pits of different size were preserved on some specimens but the flanks of these scratches did show erosion.

Among the papionins recovered in the Shungura Formation, the species *Papio* (*Dinopithecus*) *quadratirostris* has been identified from Member D to the lower part of Member G. However, Frost^34^ suggested that this species, or at least the genus *Papio*, is present from Member A to Member L. Here, only the specimens attributed to the genus *Papio* were considered, meaning that no other papionin material was included.

Two species of *Theropithecus* are represented (Appendix 1): *T*. *brumpti* (n = 42) and *T*. *oswaldi* (n = 6). It is worth mentioning that the specific attribution of many specimens of *Theropithecus* that we considered is uncertain (n = 106; see Appendix 1). *Theropithecus brumpti* has been recovered in members A to D and, starting from Member E, it is associated with *T*. *oswaldi*. Thus, specimens from the lower members can most probably be attributed to *T*. *brumpti*, but specimens from Member E to the lower part of Member G can belong to either species. It was decided to work at the generic level as a specific level attribution for these fossil taxa cannot be made with certainty on isolated teeth. However, specific differences between *T*. *oswaldi* and *T*. *brumpti* from the Shungura Formation were explored. Due to the low sample size when considering only specimens co-occurring in the same member, it was decided to compare the two species of *Theropithecus* by including all specimens assigned to the specific level, regardless of the member in which they were found (Appendix 4).

Extant and adult wild-shot specimens of *T*. *gelada* (n = 22), *P*. *hamadryas anubis* (n = 15), *P*. *hamadryas cynocephalus* (n = 18), and *P*. *hamadryas hamadryas* (n = 6) were also sampled (Appendix 1). Among the gelada samples, nine specimens come from Guassa (housed at the Authority for Research and Conservation of Cultural Heritage in Addis Ababa), an ecosystem believed to be almost intact where the behavioral and feeding ecologies of geladas, as well as the structure and composition of the vegetation have been documented^35–37^. The other specimens of geladas come from the surroundings of Debark, to the west of the Simien Mountain National Park, and from different areas not located in Ethiopia (housed in the MNHN collections in Paris, France and the Naturmuseum Senckenberg, Frankfort, Germany).

### Dental Microwear Texture Analysis

DMTA has been discussed in detail by Scott *et al*.^38,39^; see also Calandra and Merceron^40^. The molars were cleaned with cotton swabs soaked in alcohol in order to remove grit, dust, and glue residues remaining on their occlusal facets. After cleaning, dental impressions were made with a silicone material (polyvinyl siloxane ISO 4823, President Regular Body, Coltène-Whaledent Corp.). Scans were made directly from the silicon molds with a Leica DCM8 white-light scanning confocal microscope (Leica Microsystems; Fig. 2) with a ×100 long-distance lens (Numerical Aperture = 0.90; working distance = 0.9 mm), housed at the PALEVOPRIM laboratory. For each specimen, one Phase I occlusal facet (facets 1–8)^41,42^ and one Phase II facet (Facets 9 to 13)^41,42^ were scanned on either the upper or lower molars (M1, M2, M3). The choice of the molar is made based on the stage of wear (stage 3–4 *sensu* Meikle^43^, or stage 2–4 *sensu* Venkataraman *et al*.^36^). The scanning process generates 350 × 264 μm point clouds with a vertical sampling lower than 0.002 μm and a lateral sampling (x, y) of 0.129 μm. These surfaces have been saved as plu files by the LeicaScan software (Leica Microsystems). They are then opened with LeicaMap software (Mountain technology, Leica Microsystems) to remove aberrant peaks with automatic operators including a morphological filter^23^. These surfaces were leveled and a 200 × 200 μm area was selected and saved as a Digital Elevation Model (.sur) to be used for DMTA.

The four most significant dental microwear texture variables were considered: *Asfc*, *HAsfc*, *epLsar* and *Tfv*, which have been described in detail by Ungar *et al*.^44^ and Scott *et al*.^38,39^. Complexity (area-scale fractal complexity, *Asfc*) is a measure of the roughness at a given scale. Heterogeneity of complexity (heterogeneity of area-scale fractal complexity, *HAsfc*) quantifies the variation of complexity observed within a scan. *HAsfc* is obtained by subdividing the surface to a 9 × 9 grid, hence 81 cells, in which complexity is calculated. Anisotropy (*epLsar* or exact proportion of length-scale anisotropy of relief) measures the orientation concentration of surface roughness. Complexity (*Asfc*), heterogeneity of complexity (*HAsfc*) and anisotropy (*epLsar*) were calculated with Toothfrax software (www.surfract.com). Textural fill volume (*Tfv*) is the result of an algorithm that fills a surface with square cuboids of different volumes (10 and 2 µm-side cuboids). *Tfv* does not depend on the surface shape but on its finer texture. It was calculated with Sfrax software (www.surfract.com).

Among Primates, the complexity of dental microwear textures is higher for taxa consuming hard food items, such as fruits with hard exocarp or hard seeds^12,38,39^. This relationship has been shown experimentally on captive primates^45,46^ but also on captive ewes^24^. High complexity can be correlated with the consumption of possibly mechanically challenging Underground Storage Organs (USOs) that may also carry grit and dust on their surface. Although quartz grains present on the surface of some food items do scratch the enamel and contribute to tooth wear^47,48^, a recent controlled food test concluded that dust load on foods (simulating natural Harmattan wind-blown dust loads in present day western Africa) does not overwhelm the dietary signal^23^. Anisotropy (*epLsar*) is higher for species consuming blades of herbaceous monocots or mature leaves from woody dicots^12,38,39^. Similarly, based on a controlled food test on ewes, Merceron *et al*.^23^ showed that mature grasses generate significantly higher anisotropy on teeth than is generated by soft herbaceous dicots. High *HAsfc* values are linked with high variability in both size and nature of wear-causing particles, indicating a broader diet in terms of fracture properties^12,39^. Ramdarshan *et al*.^24^ showed that a mixture of foliage and seeds produces higher heterogeneity of complexity than is produced by a dietary bolus composed exclusively of herbaceous dicots.

### Statistical analyses

#### Intergeneric variations

To investigate potential differences in terms of diets between extinct *Papio* and *Theropithecus* and their extant relatives, consideration was given only to geological members for which there were at least seven specimens per genus as well as the two extant samples of *Papio* and *Theropithecus*. These geological members with substantial specimen numbers are E, F, and G (Table 1). The four dental microwear texture variables were considered (*Asfc*, *HAsfc*, *epLsar*, and *Tfv*) but only on Phase II molar facets, as these facets have been considered more significant for discriminating primates with contrasting diets^49^.

**Table 1:** Descriptive statistics of textural parameters from Phase II facets of Shungura papionins from members E, F, and G, and of their extant relatives.

			*Asfc*			*HAsfc*			epLsar (×10^−3^)			Tfv (μm^3^)		
Genera	Samples	N	m	sd	sem	m	sd	sem	m	sd	sem	m	sd	sem
*Papio*	Member E	14	**2**.**32**	2.51	0.67	**0**.**48**	0.21	0.06	2.49	1.51	0.40	**41805**	11810	3156
	Member F	10	**1**.**51**	1.08	0.34	**0**.**46**	0.20	0.06	2.55	1.77	0.56	**39148**	10144	3208
	Member G	17	**1**.**70**	1.05	0.25	**0**.**50**	0.24	0.06	1.98	1.24	0.30	**33058**	15290	3708
	*P. hamadryas anubis*	15	**1**.**46**	0.66	0.17	**0**.**58**	0.13	0.03	3.29	1.48	0.38	**40299**	9774	2524
	*P. h. cynocephalus*	18	**1**.**87**	1.35	0.32	**0**.**58**	0.17	0.04	2.90	1.67	0.39	**36491**	13376	3153
	*P. h. hamadryas*	6	**1**.**55**	0.75	0.30	**0**.**67**	0.26	0.10	3.36	2.64	1.08	**29184**	11317	4620
*Theropithecus*	Member E	8	**1**.**19**	0.19	0.07	**0**.**43**	0.11	0.04	2.05	1.42	0.50	**43795**	8646	3057
	Member F	14	**1**.**29**	0.52	0.14	**0**.**41**	0.14	0.04	2.37	1.67	0.45	**41649**	11135	2976
	Member G	16	**1**.**23**	0.56	0.14	**0**.**36**	0.06	0.02	3.18	2.39	0.60	**46263**	8861	2215
	*T. gelada*	20	**1**.**21**	0.76	0.17	**0**.**42**	0.14	0.03	4.11	1.96	0.44	**43596**	10378	2321

N: number of specimens. m: mean. sd: standard deviation. sem: standard error of the mean.

Prior to the analysis, variables were rank-transformed to avoid normality assumption violations in parametric tests^50,51^. One-way analyses of variance (ANOVAs) were performed for each parameter (Table 2). Finally, for each significant parameter, pairwise comparisons were performed using the combination of Tukey’s HSD (Honest Significant Difference) and the less conservative Fisher’s LSD (Least Significant Difference; Table 3; Appendix 3) tests.

**Table 2:** Univariate Analyses of Variance on each textural parameter, calculated on Phase II molar facets to investigate differences between extinct and extant *Theropithecus* and *Papio*.

*Asfc*					
Factor	df	SS	MS	F	p
Genus	1	10964.4	10964.4	7.1303	**0**.**009**
Sample (Genus)	8	7427.8	928.5	0.6038	0.773
Error	128	196826.4	1537.7		
***HAsfc***					
Genus	1	27684.2	27684.2	21.5552	**<0**.**001**
Sample (Genus)	8	22739.6	2842.5	2.2132	**0**.**030**
Error	128	164395.2	1284.3		
***epLsar***					
Genus	1	48.6	48.6	0.0325	0.857
Sample (Genus)	8	26188.0	3273.5	2.1897	**0**.**032**
Error	128	191353.9	1495.0		
***Tfv***					
Genus	1	12412.1	12412.1	8.2066	**0**.**005**
Sample (Genus)	8	13557.5	1694.7	1.1205	0.354
Error	128	193595.1	1512.5

**Table 3:**
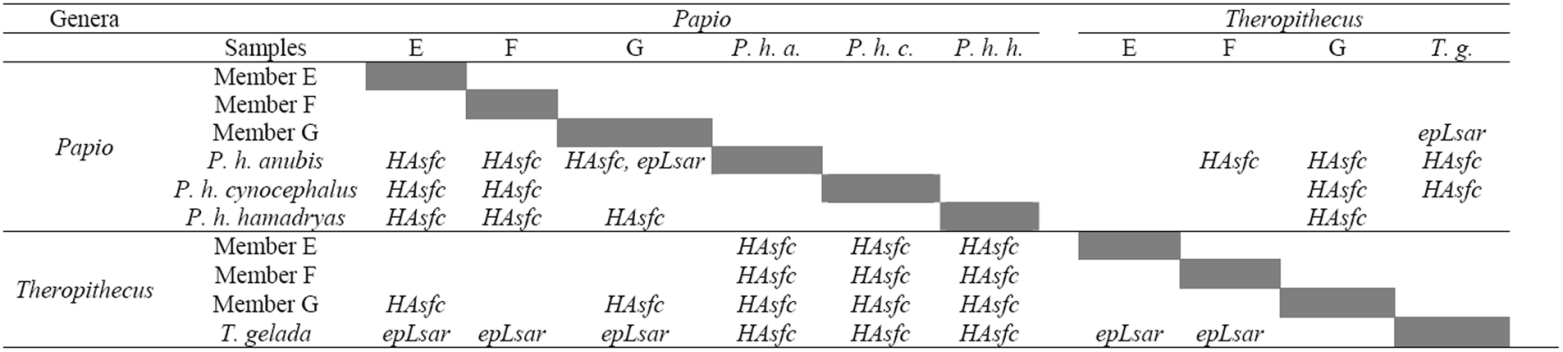
Summary of post hoc tests on texture parameters obtained from Phase II facets of extinct and extant *Theropithecus* and *Papio*. Variables written above the diagonal are significant with HSD. Variables written under the diagonal are significant with LSD.

#### Chronological variations

**Figure 3:**
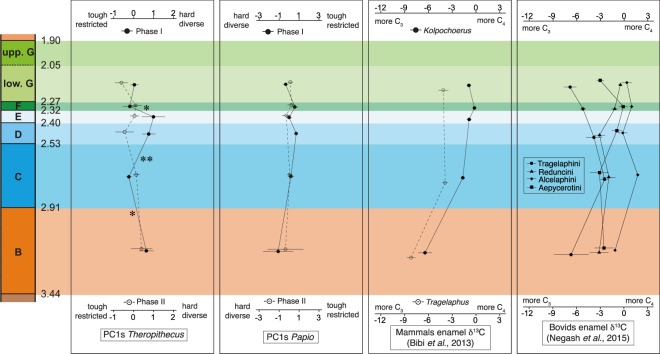
Temporal paleodietary fluctuations across the geological members of the Shungura Formation. Symbols on the curves represent means and error bars represent standard errors of means. Asterisks pinpoint significant differences on PC1 coordinates for the Phase I (Wear Textural Index) of *Theropithecus*. A single asterisk means a moderate difference (only LSD is significant) and two asterisks mean a substantial difference (both LSD and HSD are significant).

For each of the two genera (*Theropithecus* and *Papio*) and for each of the two sets of dental facets (Phase I and Phase II facets), four Principal Component Analyses (PCAs) were generated, followed by a set of ANOVAs to identify significant variations between stratigraphic samples (Appendices 6 and 7). Only the PCA on Phase I molar facets of *Theropithecus* shows significant variations along the first and the third Principal Components (PC). As for generic variations, pairwise comparisons on ranked PC1 and PC3 coordinates were performed with the tandem HSD-LSD (Appendices 8 and 9). As the coordinates along the most informative component, PC1, of the PCA conducted on Phase I facets of *Theropithecus* display significant variations across geological time, this linear combination of the four dental microwear texture parameters (Fig. 3) will be referred to as the Wear Textural Index (*WTI*).

## Results

### Differences between modern and fossil *Papio* and *Theropithecus*

The two-way nested ANOVAs (using genus and sample as factors) ran on ranked data detect variations in all surface parameters, between genera in complexity *Asfc*, heterogeneity of complexity *HAsfc* and textural fill volume *Tfv*, and between samples for heterogeneity of complexity *HAsfc* and anisotropy *epLsar* (Table 2). The samples in question were of extant species of *Theropithecus* and *Papio* along with extinct papionins grouped by genus and stratigraphic provenance.

When considering whole genera, *Papio* differ from *Theropithecus* in having significantly higher *Asfc* and *HAsfc*, and lower *Tfv*, but there is no perceptible difference for *epLsar* (Tables 1 and 2). It should be noted that this result combines both extant and extinct representatives of the two genera.

When considering differences between samples, *Theropithecus gelada* exhibit a lower *HAsfc* compared to extant *Papio* species. Extinct *Theropithecus* from members E and F differ from their extant relatives in having a moderately lower *epLsar*, whereas *Theropithecus* from G do not differ from *T*. *gelada* based on this variable (Table 3). Extinct *Papio* from members E and F differ from the three extant species in having a moderately lower *HAsfc*. *Papio* from Member G differ from *Papio hamadryas hamadryas* in having a lower *HAsfc* and from *P*. *hamadryas anubis* in having lower *HAsfc* and *epLsar*, but they do not differ from *P*. *hamadryas cynocephalus* (Table 3). When considering extinct papionins, *Papio* from members E, F, and G do not differ from *Theropithecus* from members E and F. *Papio* from members E and G show a moderately higher *HAsfc* than *Theropithecus* from Member G whereas *Papio* from Member F do not differ from *Theropithecus* from G (Table 3).

### Chronological variations

Before drawing any interpretations on the temporal dietary fluctuations of Shungura *Theropithecus*, it was important to check whether the two species identified in the formation (*i*.*e*. *T*. *brumpti and T*. *oswaldi*) had different dietary preferences that could affect the signal, as *T*. *oswaldi* was not recovered from the lowest geological members (B to D). Hence, two sets of ANOVAs were performed on each texture parameter (complexity *Asfc*, heterogeneity of complexity *HAsfc*, anisotropy *epLsar*, and textural fill volume *Tfv*) for the two types of facets (Phase I and Phase II). None of the ANOVAs resulted in a significant difference between the two species when considering all attributed specimens whatever the member in which they were found (Appendix 4).

ANOVAs performed on coordinates obtained from the four PCAs revealed that only PC1 and PC3 coordinates from the analysis on Phase I facets of *Theropithecus* show significant differences between geological members (Appendices 6 and 7). PC coordinates obtained from both Phase I and Phase II *Papio* molar facets show no variations over time. The same is true for Phase II *Theropithecus* facets that also display no variations along the stratigraphic sequence (Appendices 6 and 7). This last result might seem unexpected because both Phase I and Phase II molar facets are involved successively during the mastication of the dietary bolus, but they do not show the same trends. However, as Phases I and II molar facets involve different kinds of masticatory movements^41,42^, they may carry different dietary signals^49^.

PC1 for Phase I *Theropithecus* facets accounts for 33.2% of the total variance and is mainly explained by the complexity *Asfc* (45.5% of variance), followed by the heterogeneity of complexity *HAsfc* (37.2%), the anisotropy *epLsar* (17.2%) and the textural fill volume *Tfv* (0.1%). *Asfc*, *HAsfc*, and *Tfv* are positively correlated with PC1, whereas *epLsar* is negatively correlated with PC1 (Appendix 6). Pairwise comparisons show fluctuations of PC1 coordinates between successive geological members with one major difference between members C and D (highlighted by significant HSD, see Appendix 8). Specimens from Member D exhibit higher values than specimens from Member C. Two minor differences are also shown between B and C and between E and F, for which only LSD is significant. Specimens from Member C show lower PC1 values than specimens from Member B. Specimens from Member F display lower PC1 values than Member E specimens (Fig. 3). As *Tfv* represents a negligible amount of the variance on PC1, this principal component is mainly explained by the complexity *Asfc* and heterogeneity of complexity *HAsfc*, which are positively correlated to the axis, and by anisotropy *epLsar*, which is negatively correlated to PC1 (Appendix 6). Therefore, the previously described differences can be interpreted as a slight decrease in both *Asfc* and *HAsfc* along with a slight increase in *epLsar* from Member B to Member C, followed by a substantial increase of *Asfc* and *HAsfc* with a marked decrease in *epLsar* from Member C to Member D, and then another slight decrease in both complexity *Asfc* and heterogeneity of complexity *HAsfc* and an increase in anisotropy *epLsar* from Member E to Member F (Fig. 3).

PC3 for Phase I facets of *Theropithecus* accounts for 19.7% of the total variance and is mostly explained by heterogeneity of complexity *HAsfc* (56.6% of variance), followed by complexity *Asfc* (21.5%), textural fill volume *Tfv* (11.6%) and anisotropy *epLsar* (10.3%). Complexity *Asfc* and textural fill volume *Tfv* are positively correlated with PC3, whereas heterogeneity of complexity *HAsfc* and anisotropy *epLsar* are negatively correlated with PC3 (Appendix 6). Pairwise comparisons highlight variations of PC3 coordinates between consecutive geological members with two minor differences (shown by significant LSD, see Appendices 8 and 9) between members D and E and then between the following Member F and the lower part of Member G. Specimens from Member E exhibit a lower mean PC3 value than Member D specimens. *Theropithecus* recovered from the lower part of Member G display a higher mean PC3 value than specimens found in Member F. PC3 is negatively explained by heterogeneity of complexity *HAsfc* and anisotropy *epLsar*, and positively explained by complexity *Asfc* and textural fill volume Tfv. Hence, the previously mentioned variations can be seen as a slight increase in mean heterogeneity of complexity *HAsfc* and anisotropy *epLsar* along with a slight decrease in complexity *Asfc* and textural fill volume *Tfv* from Member D to Member E, followed by a slight decrease in means of heterogeneity of complexity *HAsfc* and anisotropy *epLsar* with a slight increase of means of complexity *Asfc* and textural fill volume *Tfv* from Member F to the lower part of Member G.

## Discussion

### Diet of extant geladas and implications for the interpretation of paleodietary proxies applied to extinct *Theropithecus*

During the wet season, the gelada diet was first thought to be almost exclusively composed of herbaceous monocots, mainly grass blades, representing more than 90% of their diet. During the dry season, they were shown to heavily rely on Underground Storage Organs (USOs), which represent more than 50% of their diet during the dry months^52^.

But most of these short-term studies had focused on populations living in disturbed areas, subject to high livestock grazing pressure, resulting in a dusty habitat dominated by short grasses^35^. Two long-term studies recently came out: one in an almost intact tall-grass afroalpine ecosystem at Guassa Plateau (Fig. 1)^35^, and the other in a disturbed area, Indetu (Fig. 1)^53^. The latter study showed that leaves of herbaceous monocotyledons represent between 38.1% in September to 71.4% of the diet in November, or 51.7% yearly. This is supplemented by USOs that compose up to 46.6% of the diet in February, or 34.8% yearly. The study has also reported that leaves of herbaceous dicots (forbs) compose up to 18.4% of the diet in April^53^, but only 7.1% of the yearly diet overall. Conversely, the study done at Guassa has shown a higher contribution of herbaceous dicots (forbs), representing 37.8% of the annual diet and an intake of herbaceous monocotyledons accounting for 56.8% of their yearly diet^35^. They also observed high seasonal dietary variations with herbaceous monocotyledon blades and inflorescences being heavily consumed during the wet season, whereas the intake of underground organs is higher for the dry season. Even during the rainiest month, herbaceous monocotyledons do not represent more than 75% of the diet, and underground organs do not account for more than 28% of the diet during the driest month^35^. As forbs represent from 20% to 60% of the gelada’s yearly diet, and because edible forbs account for only 7.4% of the ground cover at Guassa, the authors considered forbs as a preferred food source^35^. Conversely, Underground Storage Organs, the consumption of which is negatively correlated with rainfall, are considered as fallback foods (FBFs)^35,36,54–57^. Because extant *Theropithecus* show broader seasonal variations in their diet than previously thought, special attention should be paid to seasonal consumption of mechanically challenging FBFs, notably due to the potential rapid overprinting of wear marks on dental facets^45,46,58^. USOs consumed by Guassa geladas as fallback foods during the dry season may not be particularly hard but they are covered in grit^36^. However, geladas have been observed to clean USOs with their hand before ingestion. Also, the tunic of corms/tubers is spat out, probably to avoid grit and limit tooth wear^35^. The observations from Fashing *et al*.^35^ on intact ecosystems compared to the studies on disturbed areas^52,53^ raise one important potential bias of uniformitarian approaches^19^. As the distribution area of extant geladas overlaps anthropic habitats shaped by millennia of tree felling, pastoralism and agriculture, its dietary habits should not be directly transposed onto its extinct relatives spread across Africa during the Plio-Pleistocene.

The specific monocot-dominated diet of geladas was also proposed for its extinct relatives, *T*. *brumpti* and *T*. *oswaldi*, based on 2D dental microwear studies and on stable carbon isotope studies^13–19,37,59,60^. Among these, Cerling *et al*.^16^ examined stable carbon isotopes analyses on the enamel of *T*. *brumpti* and *T*. *oswaldi* (n = 41) from the Turkana Basin and from Olorgesaillie in southern Kenya. *T*. *brumpti* specimens exhibit a mean δ^13^C value indicating a diet comprising between 55% and 75% of C_4_ plants, whereas *T*. *oswaldi* specimens show a diet even more focused on C_4_ plants, representing between 70% and 90% of its consumption. The present study did not detect any significant difference between the two species (Appendix 5). DMTA might reach its limits to discriminate two grass-eating monkeys, notably taking into account the low sample size regarding *T*. *oswaldi*. Alternatively, it can be hypothesized that the two species of *Theropithecus* shared food resources with similar mechanical properties between 2.5 and 2.0 Ma, an interval of time not explored by Cerling *et al*.^16^. It is also worth pointing out here that many herbaceous monocots are not C_4_ but C_3_ plants. As recalled by Souron^51^, the C_3_ component in mammal paleodiets is frequently assumed to come from trees, bushes or shrubs, and C_4_ from herbaceous monocots. However, growing evidence indicates that, during the Plio-Pleistocene, C_3_ herbaceous monocots such as grasses, sedges and rushes were more abundant in the tropical lowlands of Africa than they are today^61–64^. Therefore, the C_3_ component of their diet can instead come from the herbaceous layer, with either herbaceous monocots or dicots (forbs), rather than from woody dicots. Of note is the fact that, although herbaceous monocots in modern African tropical lowland ecosystems are mainly C_4_, this is not the case in the Highlands inhabited by extant geladas, where C_3_ monocotyledons represent the dominant part of the herbaceous layer^35,65–67^. Although several sets of enamel stable carbon isotopes analyses on extinct specimens of *Theropithecus* point to a mixed C_3_/C_4_^18^ to dominant C_4_ diet^16^, it is surprising that there is hitherto only one stable carbon isotope data published on modern gelada, which is below −10‰^68^. There is no doubt that supplementary analysis would confirm the very low proportion of C_4_ because the Highlands where the extant geladas live are too high in altitude to favor C_4_ grasses at the expense of C_3_ ones.

DMTA on extant primates^12,69,70^ and notably geladas are scarce^12,37^. Scott *et al*.^12^ showed that modern geladas have low complexity and high anisotropy, suggesting the consumption of tough plants, presumably herbaceous monocots. Later, Shapiro *et al*.^37^ presented slightly different textures. The extant geladas they analyzed show lower values in anisotropy compared to the specimens sampled by Scott *et al*.^12^. This fits with the lower amount of herbaceous monocots consumed by the Guassa population studied by Shapiro *et al*.^37^. The sample of extant geladas used here also comes partly from Guassa and as such the values of anisotropy and complexity that were obtained are similar to the values found by Shapiro *et al*.^37^. This supports the conclusions of Fashing *et al*.^35^ that this species has a broader diet including fewer leaves of herbaceous monocots than previously thought.

It is not unexpected to find such values of anisotropy in the extinct specimens of *Theropithecus* from the Shungura Formation. Moreover, it is worth mentioning that the extant sample of geladas does not differ from the samples of modern baboons in anisotropy (*epLsar*) but rather in complexity (*Asfc*) and in heterogeneity of complexity (*HAsfc*; Tables 1 and 3).

### Dietary differences between terrestrial papionins from the Shungura Formation

The only enamel stable carbon isotope analysis exploring niche partitioning among terrestrial papionins was conducted by Lee-Thorp *et al*.^13^ on *T*. *darti* and *P*. *robinsoni* from Swartkrans in South Africa. The authors detected that the former species included high proportions of C_4_ vegetation in its diet, presumably herbaceous monocots, whereas the latter included only C_3_ vegetation. The present study also explores differences between species of *Theropithecus* and *Papio* in the Shungura Formation.

Three geological members (E, F, and G) provide enough preserved material to compare differences in dental microwear textures between the two genera (Appendix 1b).

As expected due to their different diets, *Theropithecus gelada* and all extant *Papio* species considered here differ from one another. Nonetheless, although extant *Papio* exhibit a higher heterogeneity of complexity, they do not differ from *T*. *gelada* when considering anisotropy. This was unexpected given the higher consumption of tough herbaceous monocots by the latter taxon. Hence, from a mechanical perspective, extant *Theropithecus* and *Papio* only differ in size and variability of wear-causing particles and therefore by a broader diet for *Papio*.

*Theropithecus* from members E and F, having lower anisotropy than their extant relatives, probably ate fewer tough herbaceous monocots and maybe more herbaceous dicots, whereas *Theropithecus* from Member G might have had a similar diet to *T*. *gelada* from Guassa^35^.

*Papio* from members E and F show a lower heterogeneity of complexity than the three extant species. This can be interpreted as a more restricted diet for the two samples of extinct *Papio* or a seasonal bias in the fossil records where more specimens died at a given season than at another one. Conversely, *Papio* from Member G do not differ from *P*. *hamadryas cynocephalus*, but probably had a more restricted diet than *P*. *hamadryas anubis* and *P*. *hamadryas hamadryas*, as well as incorporating fewer tough plants than *P*. *hamadryas cynocephalus*.

The main goal of this analysis was to track potential ecological segregation between extinct *Papio* and *Theropithecus* recovered in the Shungura Formation. Although the two genera do not differ in members E and F, *Papio* from Member G differ from the sympatric *Theropithecus* in having a higher heterogeneity of complexity, reflecting a broader diet in terms of mechanical properties of the food.

When considering the results and the previous isotope analyses, it is possible to conclude that the ecological segregation between the extant geladas and baboons is nested in their evolutionary history, at least from 2.27 Ma onwards. This corresponds to the very base of Member G.

### Temporal variations in the diets of terrestrial papionins from the Shungura Formation

Phase I facets of *Theropithecus* show significant differences between geological members. As Phase I molar facets contribute to the shearing of food items, it can be hypothesized that changes in dental microwear textures on Phase I facets over time would reflect the abundance of tough vegetation, possibly herbaceous monocots in the diet of *Theropithecus*. Considering *Papio*, neither Phase I nor Phase II molar facets show differences in microwear textures, suggesting no significant changes in feeding resources over time. This is not unexpected as extant *Papio* has a varied diet and thus changes in environmental conditions would not impact the whole food stock, but only a portion of it. Conversely, environmental changes would have higher impacts on species like the modern geladas with a narrower range of foods most of them being issued from the herbaceous layer.

Here, instead of exploring variations through geological times for all of the surface texture parameters (see Materials and Methods), a synthetic linear combination was computed to summarize the entire dental microwear texture into a single attribute, rendering fluctuations through time easier to interpret. The *WTI* (first PC of the PCA computed from textures on Phase I molar facets of *Theropithecus*) shows significant variations, suggesting fluctuations in the dietary habits of those terrestrial papionins (Fig. 3; Appendix 8).

The first putative dietary change is observed between members B and C at about 2.91 Ma (Fig. 3). *Theropithecus* from Member B potentially had a more diverse diet than their relatives from Member C. The former might have consumed more hard and brittle foods, which are more mechanically challenging, such as USOs, stems or seeds of herbaceous plants. Geladas at Guassa have been shown to sometimes consume invertebrates, so such food items might also have been eaten by the specimens of *Theropithecus* recovered in Member B that were analyzed here. Conversely, the specimens from Member C probably had a more restricted diet including fewer hard but more tough food items. In the herbaceous layer, such mechanical properties are notably found for the blades of herbaceous monocots, which are soft and tough^36^, so *Theropithecus* recovered in Member C may have ingested more of these plants. Cerling *et al*.^20^ analyzed the enamel stable carbon isotope of *T*. *brumpti* and *T*. *oswaldi* from the Turkana Basin and from Olorgesaillie in southern Kenya. In the current study, the only time window in common with the latter study runs from 3.44 Ma (the base of Member B) to 2.53 Ma (the top of Member C), in which only *T*. *brumpti* occurs. Cerling *et al*.^20^ found that *T*. *brumpti* includes from 55% to 75% of C_4_ plants in its diet. This is consistent with the results here. However, they did not check for fine temporal dietary fluctuations during this time interval.

The second piece of putative dietary shift evidence is the most significant of all the fluctuations observed (Fig. 3). It happened between the geological members C and D at about 2.53 Ma. Specimens of *Theropithecus* from Member D had a clearly more diverse diet than the earlier individuals from Member C, and also ate more hard and likely fewer tough foods. When considering food items available both above and underground, *Theropithecus* specimens from Member D probably had a similar diet to their relatives from Member B as they might have eaten a variety of mechanically challenging foods such as seeds, stems, and USOs from both herbaceous dicots and monocots, as well as invertebrate prey.

The third probable dietary shift happened at about 2.32 Ma, between members E and F (Fig. 3). *Theropithecus* from Member F likely ingested less quantities of hard foods but more of tougher foods, and their diet was also more restrictive than their relatives from Member E. Similarly to Member C specimens, these *Theropithecus* may have eaten more herbaceous monocot blades, with fewer hard items such as seeds, stems, or USOs. They probably also ate some leaves of herbaceous dicots.

### Environmental proxies and diets of *Theropithecus*

In order to make inferences on vegetal resources available during the Plio-Pleistocene along the ancestral Omo River, it is essential to combine proxies. The aim here is to test whether the fluctuations in dental microwear textures observed on *Theropithecus* reflect behavior, or mirror environmental changes occurring at the regional scale. It can be supposed that ephemeral local changes in available resources potentially impacting few individuals are unlikely to be detectable through a random sampling of fossil specimens. This is true whatever the proxy used, either from dental microwear texture or from stable isotope analyses. Only long-lasting regional changes that deeply affect feeding resources will potentially be noticeable from these paleodietary proxies.

DMTA on shearing facets of Shungura *Theropithecus* indicated several potential dietary shifts. The first one occurred around 2.91 Ma, between members B and C (Fig. 3). In the latter member, the diet of *Theropithecus* may have incorporated more blades of herbaceous monocots (Fig. 3). By conducting a high-resolution serial isotopic analysis of the ever-growing canines of two hippopotamid specimens from Shungura units B-9 (ca. 3 Ma) and C-9 (ca. 2.5 Ma), Souron *et al*.^71^ detected an environmental shift that could be linked with the dietary change observed in *Theropithecus*. In addition to higher δ^13^C values, the specimen from the later unit displays a higher intra-tooth amplitude of the δ^18^O values than the older specimen^71^. This may reflect a higher seasonality of rainfall in Member C^71^. Yet, these results should be considered with great caution, given the number of specimens involved (n = 2). Using enamel stable carbon isotopes on one suid genus (*Kolpochoerus*) and one bovid genus (*Tragelaphus*), Bibi *et al*.^72^ showed a trend towards the incorporation of more C_4_ plants between units B-10 and C-4/C-5 (2.97 Ma-2.74 Ma). Another isotopic study conducted by Negash *et al*.^73^ showed that, in members B and C, reduncins, alcelaphins and aepycerotins all included C_4_ plants in their diet to different degrees. However, both DMTA and the molar mesowear score do not support a dietary shift towards more grazing, but rather constant browsing habits for tragelaphins in members B and C^74^. This apparent discrepancy reflects either the consumption of soft, C_4_-growing monocotyledons that are less tough and poorer in biosilica, or the consumption of C_4_ dicots such as Amaranthaceae *sensu lato* (including Chenopodiaceae) that are regionally abundant, especially increasing through the 3.4 Ma-2.6 Ma interval^61,63^. Some of the Amaranthaceae are C_4_ and succulent dicots^75^ and were reported in the pollen fossil records in the Shungura Formation by Bonnefille and Dechamps^76^. Although pollen assemblages might be subject to different biases, these authors concluded that the vegetation in unit B-10 (ca. 3 Ma) comprises the highest proportion of arboreal taxa (27.7% of harvested pollen) along with the lowest frequency of grass pollen, which still represents no less than 43% of collected pollen^76^. The authors identified a shift in the stratigraphic unit C-9 (ca. 2.5 Ma) towards a higher proportion of grass pollen (60%) along with the decrease in abundance of montane forest taxa, which represent 21% of the collected pollens in the stratigraphic unit C-7 and no more than 9.5% in C-9^76^. Dental microwear textures of *Theropithecus* are congruent with the presence of C_4_ plant-eating ungulates in members B and C, a significant shift around 2.8 Ma in dietary habits towards further grazing habits of certain mammals^71–73^, as well as also being congruent with the pollen evidence. This would support the occurrence of a regional environmental shift around 3 Ma-2.5 Ma characterized by the spread of more open habitats, such as grasslands or shrub land, at the expense of the tree cover.

The second and main putative dietary shift inferred from dental microwear textures of *Theropithecus* occurred between members C and D at 2.53 Ma. This is characterized by a probable higher consumption of mechanically challenging foods (Fig. 3). Because USOs are predominantly consumed during the dry season by extant geladas at Guassa, though representing no more than 28% of the diet during the driest month^35^, and because these USOs account for a higher portion of the diet, frequently more than 50%^52^, in highly seasonal habitats^53^, this dietary shift could be explained by a harsher seasonality in Member D.

Dental microwear textures of *Theropithecus* from members E and F highlight another potential dietary change around 2.32 Ma, with a diet more focused on blades from herbaceous monocots for specimens recovered in Member F (Fig. 3). Negash *et al*.^73^ invoked a shift towards higher δ^13^C values when considering all bovid tribes except for tragelaphins, which displayed the opposite trend. This implies a greater C_4_ intake between Member D (2.53 Ma-2.4 Ma) and Member F (2.32 Ma-2.27 Ma) deposits. The fact that herbaceous monocots seem to be more consumed by both *Theropithecus* and most of the bovids from Member F suggests that these plants were more abundant in the milieu. It is worth mentioning that based on the combination of DMTA and the stable carbon isotopic composition on tooth enamel^74^, the tragelaphins kept browsing during Member F deposition, suggesting a more significant ecological segregation with reduncins. What is surprising is that Bonnefille and Dechamps^76^ have described the Member E as “*an almost treeless vegetation […] evidenced by the pollen spectrum of E-4*” (p. 206). Interestingly, in F-1, fewer grasses have been identified than in the previous member and there is a slight return of montane forest taxa at 8% and species restricted to riverine forests^76^. However, even though fewer grass pollens were recovered in the stratigraphic unit F-1, their proportion of 57% is higher than in stratigraphic unit B-10 at 43% and equivalent to unit C-9 at 60%. Therefore, the herbaceous layer probably comprised more monocots in Member F than in Member B.

During the transition from Member F to the lower part of Member G, aepycerotins and tragelaphins both show a decrease in mean δ^13^C, implying less C_4_ consumption^73^. Bibi *et al*.^72^ did not detect such a shift when considering tragelaphins from various sites along the lower part of the Member G. Based on direct evidence from pollens gathered by Bonnefille and Dechamps^76^, in Member G grass pollen accounts for only 53% of the pollinic spectrum and the frequency of riparian forest taxa is the highest, indicating a wetter environment. The *WTI* on *Theropithecus* does not reflect significant changes in diet between Member F and the lower part of Member G (Fig. 3). However, the PC3 coordinates (mainly explained by *HAsfc* and *Asfc*; Appendix 6) of the analysis vary significantly with a more homogenous, harder, and less tough diet in Member G than in Member F (Appendices 9 and 10).

## Conclusions

The present study is the first to apply DMTA on a broad sample of terrestrial papionins, more precisely of *Papio* and *Theropithecus* found in the Shungura Formation. Two aspects were evaluated: differences in diet between contemporaneous extinct *Papio* and *Theropithecus* in comparison to the diets of their extant relatives, and potential dietary fluctuations along the stratigraphic sequence.

The results here show that, even by 2.27 Ma onwards (lower part of Member G), extinct representatives of the genera *Papio* and *Theropithecus* already differed in diet, paralleling the situation showed for their extant relatives. The former probably had a more mixed diet including a greater number of hard food items. When performing a linear combination of all the texture parameters, neither the Phase I nor Phase II molar facets of *Papio* nor the Phase II facets of *Theropithecus* displayed temporal variations along the Shungura Formation, from Member B to lower part of Member G. Conversely, the Phase I facets of *Theropithecus* specimens suggested several dietary shifts along the stratigraphic sequence. Two events were recognized at about 2.91 Ma (between Member B and Member C) and 2.32 Ma (between Member E and Member F) respectively, possibly indicating higher intakes of herbaceous monocots. These two shifts are separated by an inverse trend at about 2.53 Ma (between Member C and Member D), characterized by a higher reliance on mechanically challenging foods.

Together with other environmental proxies, these potential dietary variations complement the understanding of environmental changes that happened during this period. Throughout this time span, terrestrial cercopithecines may have shared habitats with early hominins. The dietary habits of early human ancestors are still a matter of debate. The contribution of animal matter and challenging foods, such as USOs, silica-bearing vegetation, hard seeds, and the contribution of exogenous particles are the subject of discussion among paleoanthropologists. Beyond the differences in molar morphology that would limit direct comparisons of dental microwear textures, the authors here support the idea that the terrestrial cercopithecines analyzed in the present study constitute an appropriate model to best interpret the niche partitioning between extinct hominins.

## Electronic supplementary material

APPENDICES
