# Supplementary material for: Dietary niches of terrestrial cercopithecines from the Plio-Pleistocene Shungura Formation, Ethiopia: evidence from Dental Microwear Texture Analysis
Source: Sci Rep. 2018 Sep 19;8:14052. doi: 10.1038/s41598-018-32092-z (PMC6145942; doi:10.1038/s41598-018-32092-z)
Supplement: Supplementary file 1 — APPENDICES [file 41598_2018_32092_MOESM1_ESM.docx]

APPENDICES

Dietary niches of terrestrial cercopithecines from the Plio-Pleistocene Shungura Formation, Ethiopia: evidence from Dental Microwear Texture Analysis

Florian Martin ^1^, Chris-Alexander Plastiras ^2^, Gildas Merceron ^1*^, Antoine Souron ^3^, Jean-Renaud Boisserie ^1, 4^

APPENDICES **-** TABLE OF CONTENTS

**Appendix 1a:** Raw data of Phase I molar facets for extant and extinct *Theropithecus* and *Papio*

**Appendix 1b:** Raw data of Phase II molar facets from extant and extinct *Theropithecus* and *Papio*

**Appendix 2:** Descriptive statistics of microwear texture parameters on both Phase I and Phase II of extinct papionins from Shungura Formation

**Appendix 3:** Post hoc tests of microwear texture parameters obtained on Phase II molar facets of extant and extinct *Theropithecus* and *Papio*

**Appendix 4:** Descriptive statistics of microwear texture parameters on both Phase I and Phase II facets of *T. brumpti* and *T. oswaldi*

**Appendix 5:** Univariate Analyses of Variance of each texture parameter on Phase I and Phase II molar facets of *Theropithecus* *brumpti* and *T. oswaldi*

**Appendix 6:** Weight of each principal component and contribution of each variable to the axes for the four PCAs performed on Phase I and Phase II molar facets of extinct *Theropithecus* and *Papio*

**Appendix 7:** ANOVAs ran on the four principal components of each PCA performed on Phase I and Phase II molar facets of extinct *Theropithecus* and *Papio*

**Appendix 8:** Between-members pairwise comparisons of PC1 coordinates on Phase I molar facets of *Theropithecus*

**Appendix 9:** Between-members pairwise comparisons of PC3 coordinates on Phase I molar facets of *Theropithecus*

**Appendix 10:** Temporal fluctuations in dental microwear textures on Phase I and Phase II molar facets of extinct *Theropithecus* and *Papio* from the Shungura Formation

**Appendix 1a:** Raw data of Phase I molar facets for extant and extinct Theropithecus and Papio

| Specimen number | Taxon | Sample | *Asfc* | *HAsfc_81 cells_* | *epLsar_1.8 μm_* (× 10^-3^) | *Tfv* |
| --- | --- | --- | --- | --- | --- | --- |
| L 44-20a | *Papio* sp. | *Papio* G | 0.771 | 0.341 | 2.749 | 27393.2 |
| L 627-240 | *Papio* sp. | *Papio* G | 2.700 | 0.469 | 0.272 | 43307.8 |
| L 628-105 | *Papio* sp. | *Papio* G | 1.446 | 0.338 | 1.446 | 38661.9 |
| L 628-231 | *Papio* sp. | *Papio* G | 1.735 | 0.971 | 0.733 | 6928.3 |
| L 628-99 | *Papio* sp. | *Papio* G | 1.213 | 1.085 | 2.593 | 29951.4 |
| OMO 143-1972-10 | *Papio* sp. | *Papio* G | 3.152 | 0.354 | 0.879 | 33575.4 |
| OMO 195-1973-995 | *Papio* sp. | *Papio* G | 1.221 | 0.300 | 1.232 | 7674.4 |
| OMO 215-1973-2559 | *Papio* sp. | *Papio* G | 3.163 | 0.530 | 0.557 | 47140.0 |
| OMO 243-1973-4839 | *Papio* sp. | *Papio* G | 0.625 | 0.520 | 2.346 | 36666.4 |
| OMO 254-1973-5158 | *Papio* sp. | *Papio* G | 1.283 | 0.327 | 0.798 | 34742.3 |
| OMO 254-1973-5159 | *Papio* sp. | *Papio* G | 2.369 | 0.414 | 2.636 | 36439.9 |
| OMO 75/S-1970-1284 | *Papio* sp. | *Papio* G | 1.613 | 0.267 | 1.517 | 33497.9 |
| OMO 75/S-1970-71 | *Papio* sp. | *Papio* G | 1.743 | 0.603 | 2.823 | 38060.3 |
| OMO Sh 1-1970-126 | *Papio* sp. | *Papio* G | 3.233 | 1.173 | 1.236 | 41504.0 |
| P 116 | *Papio* sp. | *Papio* G | 0.777 | 0.508 | 1.146 | 34522.3 |
| L 133-7 | *Papio* sp. | *Papio* F | 1.947 | 0.350 | 1.434 | 6928.3 |
| L 215-20 | *Papio* sp. | *Papio* F | 0.640 | 0.635 | 0.555 | 20443.4 |
| L 28-151 | *Papio* sp. | *Papio* F | 0.467 | 0.595 | 1.750 | 36879.6 |
| L 398-1303 | *Papio* sp. | *Papio* F | 1.206 | 0.634 | 6.773 | 51208.5 |
| L 398-141 | *Papio* sp. | *Papio* F | 0.740 | 0.313 | 1.421 | 15029.0 |
| OMO 167-1973-641 | *Papio* sp. | *Papio* F | 1.451 | 0.334 | 2.063 | 47322.5 |
| OMO 33-1969-346 | *Papio* sp. | *Papio* F | 0.760 | 0.393 | 1.905 | 213.2 |
| OMO 33-1969-385 | *Papio* sp. | *Papio* F | 0.608 | 0.457 | 3.187 | 19399.1 |
| OMO 33-1970-2520 | *Papio* sp. | *Papio* F | 1.053 | 0.385 | 3.851 | 26647.1 |
| OMO 33-1970-2540 | *Papio* sp. | *Papio* F | 2.917 | 0.434 | 1.622 | 38748.3 |
| OMO 33-1972-30 | *Papio* sp. | *Papio* F | 3.576 | 0.759 | 0.955 | 36743.6 |
| OMO 79-10003 | *Papio* sp. | *Papio* F | 0.596 | 0.396 | 4.818 | 29571.6 |
| L 338a-2 | *Papio* sp. | *Papio* E | 3.520 | 0.841 | 1.467 | 41257.4 |
| L 4-13a | *Papio* sp. | *Papio* E | 2.185 | 0.322 | 2.765 | 20985.6 |
| L 4-14f | *Papio* sp. | *Papio* E | 0.775 | 0.353 | 0.167 | 29738.2 |
| L 82-7a | *Papio* sp. | *Papio* E | 2.522 | 1.092 | 3.823 | 30880.9 |
| OMO 166-1973-709 | *Papio* sp. | *Papio* E | 0.681 | 0.364 | 2.941 | 48738.8 |
| OMO 166-1973-712 | *Papio* sp. | *Papio* E | 1.030 | 0.318 | 3.035 | 23662.6 |
| OMO 166-1973-808 | *Papio* sp. | *Papio* E | 0.959 | 0.674 | 1.369 | 23080.7 |
| OMO 50/F-1970-2327 | *Papio* sp. | *Papio* E | 0.913 | 0.282 | 2.017 | 33255.6 |
| OMO 57/5-1972-186 | *Papio* sp. | *Papio* E | 1.871 | 0.351 | 0.471 | 22490.2 |
| OMO 57/5-1972-187 | *Papio* sp. | *Papio* E | 1.019 | 0.371 | 4.609 | 5755.8 |
| OMO 57/5-1972-63 | *Papio* sp. | *Papio* E | 1.509 | 0.303 | 0.895 | 24966.8 |
| OMO 58-1972-3 | *Papio* sp. | *Papio* E | 2.455 | 0.162 | 0.807 | 44013.7 |
| OMO 71-1970-35 | *Papio* sp. | *Papio* E | 2.273 | 0.389 | 0.968 | 35320.1 |
| OMO 92-10013 | *Papio* sp. | *Papio* E | 3.310 | 0.417 | 0.822 | 33682.0 |
| OMO 92-1970-244 | *Papio* sp. | *Papio* E | 2.033 | 0.296 | 1.634 | 36228.1 |
| L 9-64 | *Papio* sp. | *Papio* D | 1.451 | 0.399 | 4.256 | 40241.5 |
| F 166-1 | *Papio* sp. | *Papio* C | 0.721 | 0.414 | 2.855 | 26114.2 |
| L 194-10002 | *Papio* sp. | *Papio* C | 3.358 | 0.414 | 0.777 | 27393.2 |
| L 51-56 | *Papio* sp. | *Papio* C | 1.179 | 0.611 | 2.173 | 31844.7 |
| OMO 18-1968-2254 | *Papio* sp. | *Papio* C | 0.936 | 0.712 | 3.293 | 37801.8 |
| OMO 18-1972-40 | *Papio* sp. | *Papio* C | 0.656 | 0.386 | 1.734 | 34408.4 |
| OMO 84-1969-486 | *Papio* sp. | *Papio* C | 1.575 | 0.479 | 3.010 | 59392.2 |
| P 791-11 | *Papio* sp. | *Papio* C | 1.046 | 0.341 | 4.151 | 53207.2 |
| L 795-2 | *Papio* sp. | *Papio* B | 1.732 | 0.826 | 0.814 | 36660.9 |
| OMO 3/O-10048 | *Papio* sp. | *Papio* B | 0.408 | 0.370 | 1.394 | 12364.3 |
| OMO 30-1970-6 | *Papio* sp. | *Papio* B | 6.147 | 0.373 | 0.773 | 46101.9 |
| MCA 442 | *Theropithecus gelada* | *T. g.* | 0.580 | 0.277 | 3.634 | 41515.0 |
| MCA 443 | *Theropithecus gelada* | *T. g.* | 0.524 | 0.593 | 4.218 | 23236.3 |
| MCA 444 | *Theropithecus gelada* | *T. g.* | 0.677 | 0.496 | 1.862 | 25881.3 |
| MCA 601 | *Theropithecus gelada* | *T. g.* | 0.734 | 0.448 | 2.417 | 41889.3 |
| MCA 631 | *Theropithecus gelada* | *T. g.* | 0.527 | 0.279 | 1.784 | 17054.2 |
| MCA 642 | *Theropithecus gelada* | *T. g.* | 0.517 | 0.271 | 4.014 | 29295.3 |
| MCA 661 | *Theropithecus gelada* | *T. g.* | 0.757 | 0.372 | 3.656 | 48147.5 |
| MCA 662 | *Theropithecus gelada* | *T. g.* | 0.481 | 0.293 | 3.680 | 25184.1 |
| MNHN-1904-161 | *Theropithecus gelada* | *T. g.* | 0.542 | 0.667 | 1.122 | 25581.2 |
| MNHN-1904-174 | *Theropithecus gelada* | *T. g.* | 1.897 | 0.836 | 2.558 | 55872.9 |
| MNHN-1969-448 | *Theropithecus gelada* | *T. g.* | 0.874 | 0.338 | 5.061 | 26300.8 |
| MNHN-1969-449 | *Theropithecus gelada* | *T. g.* | 1.115 | 0.616 | 2.579 | 52338.1 |
| MNHN-1969-450 | *Theropithecus gelada* | *T. g.* | 0.490 | 0.391 | 3.571 | 40269.4 |
| MNHN-1969-451 | *Theropithecus gelada* | *T. g.* | 0.844 | 0.397 | 5.885 | 32365.8 |
| MNHN-1969-452 | *Theropithecus gelada* | *T. g.* | 0.871 | 0.364 | 3.112 | 27073.5 |
| MNHN-1969-453 | *Theropithecus gelada* | *T. g.* | 1.286 | 0.617 | 0.783 | 37861.3 |
| MNHN-1972-360 | *Theropithecus gelada* | *T. g.* | 0.864 | 0.364 | 4.594 | 28802.1 |
| MNHN-1972-361 | *Theropithecus gelada* | *T. g.* | 0.692 | 0.318 | 6.187 | 56685.2 |
| MNHN-A1440 | *Theropithecus gelada* | *T. g.* | 0.478 | 0.339 | 1.091 | 42609.7 |
| SNG 1011 | *Theropithecus gelada* | *T. g.* | 0.807 | 0.689 | 2.751 | 40416.2 |
| SNG 16665 | *Theropithecus gelada* | *T. g.* | 0.547 | 0.416 | 2.528 | 52428.7 |
| L 830-1 | *Theropithecus brumpti* | *Theropithecus* G | 1.443 | 0.336 | 1.749 | 7994.1 |
| OMO 75/i-1970-1055 | *Theropithecus brumpti* | *Theropithecus* G | 1.080 | 0.406 | 4.232 | 38538.8 |
| OMO Sh 1-1970-124 | *Theropithecus brumpti* | *Theropithecus* G | 0.949 | 0.363 | 2.751 | 29082.6 |
| L 607-22 | *Theropithecus oswaldi* | *Theropithecus* G | 0.860 | 0.293 | 2.015 | 426.4 |
| OMO 103-1973-4401 | *Theropithecus oswaldi* | *Theropithecus* G | 1.094 | 0.276 | 1.990 | 35215.8 |
| OMO 223-1973-2870 | *Theropithecus oswaldi* | *Theropithecus* G | 1.467 | 0.241 | 1.437 | 32189.7 |
| OMO 75/i-1970-1000-1002 | *Theropithecus oswaldi* | *Theropithecus* G | 1.005 | 0.232 | 2.095 | 23691.4 |
| OMO 75/i-1970-1003 | *Theropithecus oswaldi* | *Theropithecus* G | 0.903 | 0.272 | 0.569 | 25794.4 |
| L 65-23 | *Theropithecus* sp. | *Theropithecus* G | 0.555 | 0.328 | 0.741 | 18439.8 |
| OMO 1/B-1970-1 | *Theropithecus* sp. | *Theropithecus* G | 2.863 | 0.401 | 2.656 | 25794.4 |
| OMO 141-1972-84 | *Theropithecus* sp. | *Theropithecus* G | 0.442 | 0.266 | 0.456 | 47330.3 |
| OMO 201-1973-1563 | *Theropithecus* sp. | *Theropithecus* G | 0.961 | 0.380 | 0.687 | 30968.3 |
| OMO 29-1970-1377 | *Theropithecus* sp. | *Theropithecus* G | 1.218 | 0.279 | 1.849 | 4050.4 |
| OMO 47-1973-59 | *Theropithecus* sp. | *Theropithecus* G | 0.303 | 0.347 | 1.325 | 0.0 |
| OMO 75/S-1970-1318 | *Theropithecus* sp. | *Theropithecus* G | 1.108 | 0.354 | 3.756 | 66280.2 |
| L 467-62 | *Theropithecus brumpti* | *Theropithecus* F | 0.507 | 0.316 | 3.239 | 46537.1 |
| L 66-17 | *Theropithecus brumpti* | *Theropithecus* F | 1.116 | 0.349 | 1.151 | 54253.5 |
| L 465-82a | *Theropithecus oswaldi* | *Theropithecus* F | 0.654 | 0.523 | 3.281 | 30487.5 |
| L 244-10005 | *Theropithecus* sp. | *Theropithecus* F | 0.904 | 0.441 | 2.038 | 22215.7 |
| L 398-1135 | *Theropithecus* sp. | *Theropithecus* F | 0.815 | 0.345 | 3.286 | 25262.3 |
| L 398-1701 | *Theropithecus* sp. | *Theropithecus* F | 1.381 | 0.465 | 1.432 | 23662.6 |
| L 398-2242 | *Theropithecus* sp. | *Theropithecus* F | 0.927 | 0.252 | 5.523 | 21850.6 |
| L 398-2596 | *Theropithecus* sp. | *Theropithecus* F | 0.754 | 0.406 | 2.469 | 42955.2 |
| L 398-346 | *Theropithecus* sp. | *Theropithecus* F | 0.295 | 0.314 | 4.636 | 34001.7 |
| L 398-544 | *Theropithecus* sp. | *Theropithecus* F | 0.507 | 0.520 | 4.218 | 24089.0 |
| OMO 33-1969-339 | *Theropithecus* sp. | *Theropithecus* F | 1.271 | 0.634 | 0.722 | 31198.8 |
| OMO 33-1973-5599 | *Theropithecus* sp. | *Theropithecus* F | 0.368 | 0.304 | 4.277 | 20678.2 |
| OMO 79-10021 | *Theropithecus* sp. | *Theropithecus* F | 0.675 | 0.278 | 6.079 | 31438.9 |
| L 49-1 | *Theropithecus brumpti* | *Theropithecus* E | 0.714 | 0.344 | 0.667 | 27786.2 |
| OMO 111-1972-11 | *Theropithecus brumpti* | *Theropithecus* E | 2.498 | 0.743 | 2.664 | 22953.1 |
| L 338/Y-41 | *Theropithecus* sp. | *Theropithecus* E | 0.956 | 0.354 | 2.304 | 17906.9 |
| OMO 151-1973-217 | *Theropithecus* sp. | *Theropithecus* E | 0.503 | 0.550 | 1.480 | 29205.2 |
| OMO 50-10010 | *Theropithecus* sp. | *Theropithecus* E | 0.586 | 0.642 | 0.573 | 51053.5 |
| OMO 57-4-10010 | *Theropithecus* sp. | *Theropithecus* E | 0.790 | 0.348 | 0.362 | 106.6 |
| OMO 57/4-1972-234x2 | *Theropithecus* sp. | *Theropithecus* E | 0.988 | 0.289 | 1.019 | 42876.0 |
| L 118-9b | *Theropithecus brumpti* | *Theropithecus* D | 1.264 | 0.390 | 1.704 | 32402.9 |
| L 119-5 | *Theropithecus brumpti* | *Theropithecus* D | 1.329 | 0.439 | 2.726 | 41731.9 |
| L 161-25a | *Theropithecus brumpti* | *Theropithecus* D | 1.147 | 0.402 | 0.859 | 17800.3 |
| L 227-5 | *Theropithecus brumpti* | *Theropithecus* D | 0.689 | 0.239 | 0.632 | 18972.7 |
| L 64-35 | *Theropithecus* sp. | *Theropithecus* D | 2.259 | 0.380 | 1.574 | 37656.3 |
| OMO 148-1972-14 | *Theropithecus* sp. | *Theropithecus* D | 1.369 | 0.367 | 2.218 | 57235.7 |
| L 193-17 | *Theropithecus brumpti* | *Theropithecus* C | 1.213 | 0.359 | 1.927 | 532.9 |
| L 193-32 | *Theropithecus brumpti* | *Theropithecus* C | 2.617 | 0.453 | 1.593 | 45424.1 |
| L 199-3 | *Theropithecus brumpti* | *Theropithecus* C | 0.712 | 0.329 | 2.154 | 36986.2 |
| L 199-5 | *Theropithecus brumpti* | *Theropithecus* C | 1.429 | 0.223 | 2.118 | 38478.4 |
| L 32-154 | *Theropithecus brumpti* | *Theropithecus* C | 1.468 | 0.245 | 0.511 | 22274.7 |
| L 327-13 | *Theropithecus brumpti* | *Theropithecus* C | 0.853 | 0.216 | 2.294 | 9166.6 |
| L 345-25 | *Theropithecus brumpti* | *Theropithecus* C | 0.829 | 0.278 | 3.311 | 37205.7 |
| L 345-27-31 | *Theropithecus brumpti* | *Theropithecus* C | 0.758 | 0.245 | 4.148 | 35474.3 |
| L 345-3 | *Theropithecus brumpti* | *Theropithecus* C | 0.884 | 0.272 | 2.670 | 7248.0 |
| L 345-4 | *Theropithecus brumpti* | *Theropithecus* C | 0.738 | 0.256 | 2.121 | 23487.4 |
| L 362-11 | *Theropithecus brumpti* | *Theropithecus* C | 1.544 | 0.279 | 1.992 | 23023.1 |
| L 37-16 | *Theropithecus brumpti* | *Theropithecus* C | 0.898 | 0.232 | 5.063 | 43168.3 |
| L 440-2 | *Theropithecus brumpti* | *Theropithecus* C | 1.319 | 0.231 | 2.962 | 41420.3 |
| L 70-4 | *Theropithecus brumpti* | *Theropithecus* C | 1.038 | 0.270 | 4.841 | 33255.6 |
| L 764-1 | *Theropithecus brumpti* | *Theropithecus* C | 0.716 | 0.232 | 3.025 | 34854.4 |
| L 870-1 | *Theropithecus brumpti* | *Theropithecus* C | 0.804 | 0.283 | 4.294 | 14389.4 |
| OMO 154-1973-271 | *Theropithecus brumpti* | *Theropithecus* C | 1.073 | 0.231 | 1.405 | 30056.1 |
| OMO 18-1967-143 | *Theropithecus brumpti* | *Theropithecus* C | 1.087 | 0.276 | 0.859 | 28541.0 |
| OMO 18-1968-368 | *Theropithecus brumpti* | *Theropithecus* C | 1.039 | 0.271 | 3.068 | 14922.4 |
| OMO 217-1973-4388 | *Theropithecus brumpti* | *Theropithecus* C | 2.362 | 0.292 | 0.919 | 27859.4 |
| OMO 40-1968-1405 | *Theropithecus brumpti* | *Theropithecus* C | 1.135 | 0.425 | 3.885 | 29844.8 |
| L 18-23 | *Theropithecus* sp. | *Theropithecus* C | 0.882 | 0.277 | 0.152 | 30004.8 |
| L 18-29 | *Theropithecus* sp. | *Theropithecus* C | 1.681 | 0.248 | 1.266 | 47162.4 |
| L 193-56 | *Theropithecus* sp. | *Theropithecus* C | 2.582 | 0.423 | 0.880 | 36986.2 |
| L 199-4 | *Theropithecus* sp. | *Theropithecus* C | 1.089 | 0.217 | 2.266 | 38904.8 |
| L 32-270 | *Theropithecus* sp. | *Theropithecus* C | 1.146 | 0.314 | 1.059 | 24446.7 |
| L 345-83 | *Theropithecus* sp. | *Theropithecus* C | 0.852 | 0.392 | 1.487 | 18866.2 |
| L 42-12 | *Theropithecus* sp. | *Theropithecus* C | 0.565 | 0.270 | 1.271 | 32935.8 |
| L 54-30 | *Theropithecus* sp. | *Theropithecus* C | 0.895 | 0.247 | 5.056 | 30061.1 |
| L 750-10010 | *Theropithecus* sp. | *Theropithecus* C | 0.914 | 0.281 | 1.500 | 26901.9 |
| OMO 154-10004 | *Theropithecus* sp. | *Theropithecus* C | 0.828 | 0.310 | 1.001 | 23526.3 |
| OMO 162-1973-489 | *Theropithecus* sp. | *Theropithecus* C | 0.871 | 0.307 | 0.311 | 56428.7 |
| OMO 18-1968-1053 | *Theropithecus* sp. | *Theropithecus* C | 1.076 | 0.275 | 0.921 | 31656.8 |
| OMO 18-1968-1075 | *Theropithecus* sp. | *Theropithecus* C | 0.571 | 0.335 | 4.445 | 57809.4 |
| OMO 18-1968-1105 | *Theropithecus* sp. | *Theropithecus* C | 0.590 | 0.290 | 1.021 | 20997.9 |
| OMO 18-1968-1110 | *Theropithecus* sp. | *Theropithecus* C | 0.712 | 0.663 | 0.673 | 28672.3 |
| OMO 18-1968-1126 | *Theropithecus* sp. | *Theropithecus* C | 0.604 | 0.300 | 2.030 | 27180.1 |
| OMO 18-1968-2237 | *Theropithecus* sp. | *Theropithecus* C | 0.611 | 0.387 | 3.363 | 42315.6 |
| OMO 18-1968-2249 | *Theropithecus* sp. | *Theropithecus* C | 1.308 | 0.368 | 1.141 | 27073.5 |
| OMO 18-1968-2251 | *Theropithecus* sp. | *Theropithecus* C | 0.669 | 0.343 | 1.916 | 42955.2 |
| OMO 18-1968-2255 | *Theropithecus* sp. | *Theropithecus* C | 0.473 | 0.359 | 6.516 | 43012.8 |
| OMO 18-1968-2264 | *Theropithecus* sp. | *Theropithecus* C | 0.912 | 0.350 | 1.629 | 28885.5 |
| OMO 18-1969-497 | *Theropithecus* sp. | *Theropithecus* C | 1.275 | 0.254 | 1.497 | 20997.9 |
| OMO 18-1969-510 | *Theropithecus* sp. | *Theropithecus* C | 0.693 | 0.366 | 6.645 | 32866.2 |
| OMO 18-1970-1831 | *Theropithecus* sp. | *Theropithecus* C | 0.805 | 0.285 | 4.267 | 43584.2 |
| OMO 18-1972-15 | *Theropithecus* sp. | *Theropithecus* C | 0.844 | 0.290 | 1.647 | 37945.5 |
| OMO 18-1973-2948 | *Theropithecus* sp. | *Theropithecus* C | 0.511 | 0.265 | 1.667 | 26667.2 |
| OMO 18-1974-35 | *Theropithecus* sp. | *Theropithecus* C | 1.001 | 0.233 | 4.539 | 33463.3 |
| OMO 18/sup-10036 | *Theropithecus* sp. | *Theropithecus* C | 0.750 | 0.341 | 2.735 | 20145.2 |
| OMO 18/sup-10042 | *Theropithecus* sp. | *Theropithecus* C | 0.919 | 0.221 | 3.002 | 47218.7 |
| OMO 18/sup-10044 | *Theropithecus* sp. | *Theropithecus* C | 0.877 | 0.473 | 0.763 | 3197.7 |
| OMO 18/sup-1967-1 | *Theropithecus* sp. | *Theropithecus* C | 1.164 | 0.286 | 1.123 | 25932.1 |
| OMO 3/2-10002 | *Theropithecus* sp. | *Theropithecus* C | 0.832 | 0.426 | 1.697 | 22589.4 |
| OMO 3/2-1974-336 | *Theropithecus* sp. | *Theropithecus* C | 1.045 | 0.236 | 2.329 | 11085.2 |
| OMO 3/2-1974-349 | *Theropithecus* sp. | *Theropithecus* C | 0.502 | 0.311 | 0.499 | 25261.5 |
| OMO 3/2-1974-387 | *Theropithecus* sp. | *Theropithecus* C | 0.846 | 0.319 | 2.818 | 35805.9 |
| OMO 349-10017a | *Theropithecus* sp. | *Theropithecus* C | 1.113 | 0.382 | 4.301 | 36480.2 |
| OMO 349-10046 | *Theropithecus* sp. | *Theropithecus* C | 2.667 | 0.666 | 2.269 | 45087.8 |
| OMO 40-10046 | *Theropithecus* sp. | *Theropithecus* C | 1.537 | 0.294 | 2.626 | 59172.1 |
| OMO 40-1969-442 | *Theropithecus* sp. | *Theropithecus* C | 0.422 | 0.356 | 5.848 | 48913.1 |
| OMO 56-10009 | *Theropithecus* sp. | *Theropithecus* C | 0.592 | 0.288 | 8.276 | 46649.5 |
| OMO 56-10016 | *Theropithecus* sp. | *Theropithecus* C | 0.442 | 0.443 | 0.835 | 16962.6 |
| OMO 56-10042 | *Theropithecus* sp. | *Theropithecus* C | 0.391 | 0.275 | 3.199 | 28459.1 |
| OMO 84-1970-116 | *Theropithecus* sp. | *Theropithecus* C | 0.600 | 0.326 | 7.052 | 45227.8 |
| OMO P 708-1970-2562 | *Theropithecus* sp. | *Theropithecus* C | 0.525 | 0.244 | 0.839 | 40454.2 |
| OMO P 791/N-1970-2498 | *Theropithecus* sp. | *Theropithecus* C | 0.595 | 0.409 | 1.355 | 33362.2 |
| L 1-392 | *Theropithecus* sp. | *Theropithecus* B | 0.865 | 0.306 | 0.923 | 26966.9 |
| L 1-620 | *Theropithecus* sp. | *Theropithecus* B | 1.463 | 0.364 | 1.383 | 45406.7 |
| L 1-622 | *Theropithecus* sp. | *Theropithecus* B | 3.290 | 0.435 | 3.726 | 39537.0 |
| L 387-10004 | *Theropithecus* sp. | *Theropithecus* B | 0.630 | 0.264 | 0.733 | 25474.6 |
| OMO 212-1973-1986 | *Theropithecus* sp. | *Theropithecus* B | 2.444 | 0.399 | 1.825 | 47427.2 |
| OMO 28-1968-1251 | *Theropithecus* sp. | *Theropithecus* B | 0.816 | 0.465 | 4.519 | 46312.7 |
| OMO 28-1968-1252 | *Theropithecus* sp. | *Theropithecus* B | 1.172 | 0.315 | 0.984 | 41210.8 |
| OMO 28-1968-1273 | *Theropithecus* sp. | *Theropithecus* B | 0.939 | 0.405 | 4.707 | 37996.6 |
| OMO 3/O-1968-2230 | *Theropithecus* sp. | *Theropithecus* B | 1.127 | 0.267 | 0.774 | 32816.4 |
| OMO 82-1970-101 | *Theropithecus* sp. | *Theropithecus* B | 0.918 | 0.408 | 1.273 | 26433.9 |

**Appendix 1b:** Raw data of Phase II molar facets from extant and extinct Theropithecus and Papio

| Specimen number | Taxon | Sample | *Asfc* | *HAsfc_81 cells_* | *epLsar_1.8 μm_* (× 10^-3^) | *Tfv* |
| --- | --- | --- | --- | --- | --- | --- |
| NHMB-10481 | *Papio hamadryas anubis* | *P. h. a.* | 1.349 | 0.888 | 3.453 | 37651.8 |
| SNG-1001 | *Papio hamadryas anubis* | *P. h. a.* | 2.163 | 0.608 | 2.533 | 30600.9 |
| SNG-4190 | *Papio hamadryas anubis* | *P. h. a.* | 1.554 | 0.409 | 4.959 | 49201.3 |
| SNG-4191 | *Papio hamadryas anubis* | *P. h. a.* | 2.220 | 0.579 | 1.414 | 49556.2 |
| SNG-4194 | *Papio hamadryas anubis* | *P. h. a.* | 1.602 | 0.415 | 3.463 | 45432.8 |
| SNG-4195 | *Papio hamadryas anubis* | *P. h. a.* | 2.518 | 0.604 | 3.125 | 35062.9 |
| SNG-47992 | *Papio hamadryas anubis* | *P. h. a.* | 1.697 | 0.525 | 1.148 | 41692.0 |
| SNG-47993 | *Papio hamadryas anubis* | *P. h. a.* | 0.958 | 0.531 | 6.346 | 40999.8 |
| SNG-47994 | *Papio hamadryas anubis* | *P. h. a.* | 2.179 | 0.759 | 3.937 | 41478.8 |
| SNG-5820 | *Papio hamadryas anubis* | *P. h. a.* | 1.383 | 0.432 | 0.947 | 50041.6 |
| SNG-5822 | *Papio hamadryas anubis* | *P. h. a.* | 1.785 | 0.553 | 3.076 | 50078.6 |
| MNHN 1969-441 | *Papio hamadryas anubis* | *P. h. a.* | 0.640 | 0.648 | 3.714 | 23236.3 |
| MNHN 1969-442 | *Papio hamadryas anubis* | *P. h. a.* | 0.593 | 0.518 | 4.443 | 18866.2 |
| MNHN 1969-443 | *Papio hamadryas anubis* | *P. h. a.* | 0.661 | 0.641 | 2.311 | 48675.7 |
| MNHN 1969-444 | *Papio hamadryas anubis* | *P. h. a.* | 0.578 | 0.528 | 4.511 | 41908.5 |
| SNG-1002 | *Papio hamadryas cynocephalus* | *P. h. c.* | 1.141 | 0.789 | 0.132 | 16472.3 |
| SNG-16653 | *Papio hamadryas cynocephalus* | *P. h. c.* | 4.180 | 0.800 | 3.972 | 28223.9 |
| MNHN 1977-6 | *Papio hamadryas cynocephalus* | *P. h. c.* | 1.359 | 0.343 | 2.898 | 10872.0 |
| NHMB 10482 | *Papio hamadryas cynocephalus* | *P. h. c.* | 1.463 | 0.680 | 1.017 | 30880.9 |
| NHMB 10495 | *Papio hamadryas cynocephalus* | *P. h. c.* | 1.010 | 0.296 | 1.088 | 38547.9 |
| NHMB 10497 | *Papio hamadryas cynocephalus* | *P. h. c.* | 0.941 | 0.793 | 2.784 | 29951.4 |
| NHMB 10501 | *Papio hamadryas cynocephalus* | *P. h. c.* | 1.311 | 0.584 | 2.763 | 33064.3 |
| NHMB 10502 | *Papio hamadryas cynocephalus* | *P. h. c.* | 0.984 | 0.540 | 5.068 | 45719.0 |
| NHMB 10503 | *Papio hamadryas cynocephalus* | *P. h. c.* | 2.081 | 0.655 | 3.409 | 14069.7 |
| NHMB 10504 | *Papio hamadryas cynocephalus* | *P. h. c.* | 1.645 | 0.850 | 4.110 | 55074.0 |
| NHMB 9207 | *Papio hamadryas cynocephalus* | *P. h. c.* | 2.525 | 0.715 | 2.201 | 43418.9 |
| NHMB 9256 | *Papio hamadryas cynocephalus* | *P. h. c.* | 6.358 | 0.610 | 1.499 | 38088.7 |
| ZSCM 1914-1452 | *Papio hamadryas cynocephalus* | *P. h. c.* | 1.148 | 0.398 | 1.065 | 36171.8 |
| ZSCM 1914-1455 | *Papio hamadryas cynocephalus* | *P. h. c.* | 1.925 | 0.418 | 1.873 | 38152.2 |
| ZSCM 1914-1459 | *Papio hamadryas cynocephalus* | *P. h. c.* | 1.350 | 0.415 | 3.451 | 54501.2 |
| ZSCM 1914-4015 | *Papio hamadryas cynocephalus* | *P. h. c.* | 1.285 | 0.457 | 3.375 | 51214.1 |
| ZSCM 1914-4016 | *Papio hamadryas cynocephalus* | *P. h. c.* | 1.300 | 0.656 | 4.836 | 38821.9 |
| ZSCM 1914-4017 | *Papio hamadryas cynocephalus* | *P. h. c.* | 1.653 | 0.465 | 6.675 | 53596.1 |
| MNHN 1853-438 | *Papio hamadryas hamadryas* | *P. h. h.* | 0.770 | 0.692 | 0.802 | 17885.8 |
| MNHN 1969-447 | *Papio hamadryas hamadryas* | *P. h. h.* | 1.833 | 0.677 | 1.801 | 35133.9 |
| MNHN 1972-355 | *Papio hamadryas hamadryas* | *P. h. h.* | 0.606 | 0.473 | 7.791 | 28709.7 |
| MNHN 1972-356 | *Papio hamadryas hamadryas* | *P. h. h.* | 1.969 | 0.610 | 2.354 | 31665.8 |
| MNHN 1972-357 | *Papio hamadryas hamadryas* | *P. h. h.* | 1.538 | 1.139 | 2.110 | 15600.3 |
| MNHN 1972-359 | *Papio hamadryas hamadryas* | *P. h. h.* | 2.560 | 0.419 | 5.303 | 46107.0 |
| L 44-20a | *Papio* sp. | *Papio* G | 0.842 | 0.522 | 0.948 | 26543.2 |
| L 627-240 | *Papio* sp. | *Papio* G | 0.950 | 0.405 | 1.151 | 2025.2 |
| L 628-105 | *Papio* sp. | *Papio* G | 1.193 | 0.440 | 0.697 | 31582.6 |
| L 628-231 | *Papio* sp. | *Papio* G | 2.797 | 1.015 | 1.443 | 33508.9 |
| L 628-99 | *Papio* sp. | *Papio* G | 0.589 | 0.472 | 1.177 | 35080.8 |
| OMO 143-1972-10 | *Papio* sp. | *Papio* G | 1.534 | 0.691 | 0.480 | 43594.1 |
| OMO 195-1973-995 | *Papio* sp. | *Papio* G | 1.451 | 0.315 | 2.663 | 213.2 |
| OMO 243-1973-4839 | *Papio* sp. | *Papio* G | 0.769 | 0.411 | 2.486 | 54695.4 |
| OMO 254-1972-5157 | *Papio* sp. | *Papio* G | 2.481 | 0.364 | 0.895 | 22381.7 |
| OMO 254-1973-5158 | *Papio* sp. | *Papio* G | 1.503 | 0.372 | 0.762 | 18120.0 |
| OMO 254-1973-5159 | *Papio* sp. | *Papio* G | 2.723 | 0.307 | 4.374 | 37304.8 |
| OMO 47-1970-2043 | *Papio* sp. | *Papio* G | 1.680 | 0.543 | 2.587 | 37148.6 |
| OMO 75/S-1970-1284 | *Papio* sp. | *Papio* G | 1.302 | 0.441 | 2.178 | 41626.2 |
| OMO 75/S-1970-71 | *Papio* sp. | *Papio* G | 1.902 | 1.133 | 3.858 | 38809.9 |
| OMO 9-1969-4405 | *Papio* sp. | *Papio* G | 0.549 | 0.440 | 1.527 | 40428.1 |
| OMO Sh 1-1970-126 | *Papio* sp. | *Papio* G | 1.895 | 0.299 | 4.124 | 49434.0 |
| P 116 | *Papio* sp. | *Papio* G | 4.775 | 0.416 | 2.391 | 49481.3 |
| L 133-7 | *Papio* sp. | *Papio* F | 3.423 | 0.356 | 1.262 | 34213.1 |
| L 215-20 | *Papio* sp. | *Papio* F | 0.694 | 0.364 | 4.859 | 40187.9 |
| L 28-151 | *Papio* sp. | *Papio* F | 1.287 | 0.599 | 2.594 | 44376.9 |
| L 398-1303 | *Papio* sp. | *Papio* F | 1.809 | 0.362 | 0.455 | 45839.9 |
| OMO 33-1969-346 | *Papio* sp. | *Papio* F | 0.688 | 0.302 | 4.070 | 35428.0 |
| OMO 33-1969-385 | *Papio* sp. | *Papio* F | 0.751 | 0.408 | 1.231 | 39011.4 |
| OMO 33-1970-2520 | *Papio* sp. | *Papio* F | 0.622 | 0.257 | 1.899 | 20997.9 |
| OMO 33-1970-2540 | *Papio* sp. | *Papio* F | 1.697 | 0.493 | 1.756 | 55221.0 |
| OMO 33-1972-30 | *Papio* sp. | *Papio* F | 3.360 | 0.964 | 1.565 | 48701.8 |
| OMO 79-10003 | *Papio* sp. | *Papio* F | 0.814 | 0.490 | 5.823 | 27499.8 |
| L 338a-2 | *Papio* sp. | *Papio* E | 1.660 | 0.636 | 4.611 | 46273.0 |
| L 4-13a | *Papio* sp. | *Papio* E | 1.572 | 0.351 | 4.796 | 53778.6 |
| L 4-14f | *Papio* sp. | *Papio* E | 1.092 | 0.650 | 3.801 | 28607.7 |
| L 82-7a | *Papio* sp. | *Papio* E | 1.441 | 0.496 | 4.785 | 29205.2 |
| OMO 166-1973-712 | *Papio* sp. | *Papio* E | 1.899 | 0.367 | 2.435 | 36806.8 |
| OMO 50/F-1970-2327 | *Papio* sp. | *Papio* E | 2.305 | 0.540 | 2.301 | 45029.1 |
| OMO 57/5-1972-186 | *Papio* sp. | *Papio* E | 0.887 | 0.249 | 2.441 | 51044.4 |
| OMO 57/5-1972-187 | *Papio* sp. | *Papio* E | 1.060 | 0.679 | 2.770 | 43313.3 |
| OMO 57/5-1972-63 | *Papio* sp. | *Papio* E | 2.274 | 0.470 | 1.292 | 37197.5 |
| OMO 58-1972-3 | *Papio* sp. | *Papio* E | 2.157 | 0.260 | 1.343 | 57534.9 |
| OMO 71-1969-482 | *Papio* sp. | *Papio* E | 10.917 | 1.025 | 0.839 | 49297.2 |
| OMO 71-1970-35 | *Papio* sp. | *Papio* E | 1.585 | 0.252 | 0.946 | 55960.3 |
| OMO 71-1970-37 | *Papio* sp. | *Papio* E | 1.700 | 0.469 | 2.148 | 17114.5 |
| OMO 92-10013 | *Papio* sp. | *Papio* E | 1.951 | 0.327 | 0.293 | 34108.3 |
| F 166-1 | *Papio* sp. | *Papio* C | 1.161 | 0.432 | 1.325 | 52061.8 |
| L 51-56 | *Papio* sp. | *Papio* C | 1.000 | 0.393 | 2.752 | 49088.3 |
| OMO 18-1968-1090 | *Papio* sp. | *Papio* C | 2.525 | 0.365 | 2.028 | 41403.4 |
| OMO 18-1968-2254 | *Papio* sp. | *Papio* C | 0.929 | 0.359 | 1.948 | 39110.1 |
| OMO 18-1972-40 | *Papio* sp. | *Papio* C | 3.044 | 0.477 | 2.309 | 56338.5 |
| OMO 53-1970-1241 | *Papio* sp. | *Papio* C | 1.534 | 0.326 | 0.982 | 33807.8 |
| OMO 3/O-10048 | *Papio* sp. | *Papio* B | 0.641 | 0.335 | 1.482 | 18333.2 |
| OMO 30-1970-6 | *Papio* sp. | *Papio* B | 2.861 | 0.814 | 0.757 | 56795.1 |
| MCA 442 | *Theropithecus gelada* | *T. g.* | 1.333 | 0.345 | 2.101 | 47380.1 |
| MCA 443 | *Theropithecus gelada* | *T. g.* | 1.022 | 0.450 | 1.931 | 36055.7 |
| MCA 444 | *Theropithecus gelada* | *T. g.* | 1.176 | 0.409 | 3.711 | 45232.3 |
| MCA 601 | *Theropithecus gelada* | *T. g.* | 0.769 | 0.386 | 3.420 | 42706.7 |
| MCA 631 | *Theropithecus gelada* | *T. g.* | 0.678 | 0.280 | 4.990 | 49555.3 |
| MCA 632 | *Theropithecus gelada* | *T. g.* | 0.527 | 0.290 | 2.708 | 39096.8 |
| MCA 642 | *Theropithecus gelada* | *T. g.* | 1.401 | 0.584 | 6.689 | 42257.4 |
| MCA 661 | *Theropithecus gelada* | *T. g.* | 0.837 | 0.280 | 6.122 | 26354.3 |
| MCA 662 | *Theropithecus gelada* | *T. g.* | 0.481 | 0.386 | 5.243 | 38727.2 |
| MNHN-1904-161 | *Theropithecus gelada* | *T. g.* | 2.515 | 0.706 | 2.054 | 56093.4 |
| MNHN-1904-174 | *Theropithecus gelada* | *T. g.* | 2.788 | 0.787 | 1.985 | 55635.5 |
| MNHN-1969-448 | *Theropithecus gelada* | *T. g.* | 1.602 | 0.445 | 3.611 | 67278.0 |
| MNHN-1969-449 | *Theropithecus gelada* | *T. g.* | 1.079 | 0.280 | 5.039 | 49032.4 |
| MNHN-1969-450 | *Theropithecus gelada* | *T. g.* | 1.291 | 0.583 | 2.197 | 37873.7 |
| MNHN-1969-451 | *Theropithecus gelada* | *T. g.* | 0.724 | 0.346 | 7.940 | 46940.9 |
| MNHN-1969-452 | *Theropithecus gelada* | *T. g.* | 0.765 | 0.369 | 6.417 | 25681.3 |
| MNHN-1969-453 | *Theropithecus gelada* | *T. g.* | 0.489 | 0.426 | 5.967 | 29700.6 |
| MNHN-1972-360 | *Theropithecus gelada* | *T. g.* | 3.127 | 0.412 | 0.809 | 36132.9 |
| MNHN-1972-361 | *Theropithecus gelada* | *T. g.* | 0.649 | 0.259 | 5.124 | 48777.6 |
| MNHN-A1440 | *Theropithecus gelada* | *T. g.* | 0.928 | 0.363 | 4.131 | 51415.4 |
| OMO 75/M-1971-601 | *Theropithecus brumpti* | *Theropithecus* G | 2.224 | 0.278 | 1.641 | 39759.2 |
| OMO Sh 1-1970-124 | *Theropithecus brumpti* | *Theropithecus* G | 1.385 | 0.428 | 4.058 | 45561.1 |
| OMO 103-1973-4401 | *Theropithecus oswaldi* | *Theropithecus* G | 0.984 | 0.333 | 2.131 | 41409.8 |
| OMO 223-1973-2870 | *Theropithecus oswaldi* | *Theropithecus* G | 1.572 | 0.359 | 3.218 | 46268.4 |
| OMO 75/i-1970-1003 | *Theropithecus oswaldi* | *Theropithecus* G | 1.230 | 0.346 | 2.014 | 52769.0 |
| L 65-23 | *Theropithecus* sp. | *Theropithecus* G | 1.173 | 0.431 | 5.910 | 68002.8 |
| L 67-121a | *Theropithecus* sp. | *Theropithecus* G | 0.622 | 0.250 | 5.703 | 47616.5 |
| OMO 1/B-1970-1 | *Theropithecus* sp. | *Theropithecus* G | 1.132 | 0.336 | 7.077 | 46759.3 |
| OMO 141-1972-84 | *Theropithecus* sp. | *Theropithecus* G | 0.581 | 0.320 | 0.922 | 34754.7 |
| OMO 201-1973-1563 | *Theropithecus* sp. | *Theropithecus* G | 0.710 | 0.359 | 1.578 | 52527.1 |
| OMO 323-10025 | *Theropithecus* sp. | *Theropithecus* G | 2.196 | 0.335 | 0.454 | 53232.9 |
| OMO 47-1973-59 | *Theropithecus* sp. | *Theropithecus* G | 0.536 | 0.319 | 7.670 | 56393.4 |
| OMO 75/N-1971-17 | *Theropithecus* sp. | *Theropithecus* G | 1.741 | 0.407 | 1.357 | 41364.5 |
| OMO 75/N-1971-21 | *Theropithecus* sp. | *Theropithecus* G | 0.838 | 0.480 | 4.816 | 36868.5 |
| OMO 75/S-1970-1318 | *Theropithecus* sp. | *Theropithecus* G | 1.907 | 0.383 | 0.295 | 42792.3 |
| OMO 75/Sd-1970-431 | *Theropithecus* sp. | *Theropithecus* G | 0.865 | 0.463 | 2.031 | 34132.0 |
| L 467-62 | *Theropithecus brumpti* | *Theropithecus* F | 0.920 | 0.280 | 4.002 | 43476.1 |
| OMO 76-1972-24 | *Theropithecus brumpti* | *Theropithecus* F | 0.701 | 0.471 | 1.048 | 39408.4 |
| L 241-10003 | *Theropithecus* sp. | *Theropithecus* F | 1.043 | 0.438 | 0.556 | 35116.1 |
| L 244-10005 | *Theropithecus* sp. | *Theropithecus* F | 1.737 | 0.422 | 6.334 | 62259.8 |
| L 398-1756 | *Theropithecus* sp. | *Theropithecus* F | 1.776 | 0.356 | 1.934 | 57850.5 |
| L 398-2596 | *Theropithecus* sp. | *Theropithecus* F | 0.979 | 0.451 | 1.804 | 43452.9 |
| L 398-346 | *Theropithecus* sp. | *Theropithecus* F | 0.883 | 0.318 | 1.288 | 54102.5 |
| L 52-122 | *Theropithecus* sp. | *Theropithecus* F | 1.954 | 0.424 | 3.702 | 47862.6 |
| OMO 33-1969-337 | *Theropithecus* sp. | *Theropithecus* F | 0.978 | 0.460 | 4.025 | 32506.7 |
| OMO 33-1969-339 | *Theropithecus* sp. | *Theropithecus* F | 1.995 | 0.385 | 2.720 | 46854.5 |
| OMO 33-1973-5599 | *Theropithecus* sp. | *Theropithecus* F | 1.332 | 0.819 | 1.332 | 32767.4 |
| OMO 76-1970-73 | *Theropithecus* sp. | *Theropithecus* F | 2.191 | 0.254 | 0.410 | 32379.5 |
| OMO 76-1970-74 | *Theropithecus* sp. | *Theropithecus* F | 0.681 | 0.349 | 1.227 | 28543.7 |
| OMO 79-10021 | *Theropithecus* sp. | *Theropithecus* F | 0.950 | 0.331 | 2.732 | 26504.8 |
| L 49-1 | *Theropithecus brumpti* | *Theropithecus* E | 1.142 | 0.366 | 5.066 | 41656.7 |
| OMO 169-1973-846 | *Theropithecus brumpti* | *Theropithecus* E | 0.919 | 0.497 | 1.253 | 63236.2 |
| OMO 207-1973-1781 | *Theropithecus brumpti* | *Theropithecus* E | 1.433 | 0.473 | 1.808 | 41961.0 |
| L 338/Y-41 | *Theropithecus* sp. | *Theropithecus* E | 1.336 | 0.336 | 0.877 | 42946.0 |
| OMO 151-1973-217 | *Theropithecus* sp. | *Theropithecus* E | 1.443 | 0.534 | 3.045 | 40591.4 |
| OMO 50-10010 | *Theropithecus* sp. | *Theropithecus* E | 1.061 | 0.362 | 2.167 | 33793.1 |
| OMO 57-4-10010 | *Theropithecus* sp. | *Theropithecus* E | 1.070 | 0.598 | 1.040 | 46648.7 |
| OMO 57/4-1972-234x2 | *Theropithecus* sp. | *Theropithecus* E | 1.100 | 0.270 | 1.134 | 39524.1 |
| L 161-25a | *Theropithecus brumpti* | *Theropithecus* D | 1.168 | 0.326 | 1.652 | 47207.0 |
| L 19-10 | *Theropithecus brumpti* | *Theropithecus* D | 0.906 | 0.307 | 3.107 | 46622.1 |
| L 227-5 | *Theropithecus brumpti* | *Theropithecus* D | 1.303 | 0.323 | 1.919 | 36189.7 |
| L 227-6 | *Theropithecus brumpti* | *Theropithecus* D | 1.000 | 0.411 | 4.723 | 39541.5 |
| L 64-35 | *Theropithecus* sp. | *Theropithecus* D | 0.586 | 0.411 | 7.709 | 45568.4 |
| L 9-133 | *Theropithecus* sp. | *Theropithecus* D | 1.237 | 0.513 | 1.954 | 34027.3 |
| L 193-32 | *Theropithecus brumpti* | *Theropithecus* C | 1.804 | 0.337 | 4.993 | 38837.0 |
| L 199-3 | *Theropithecus brumpti* | *Theropithecus* C | 1.188 | 0.379 | 1.271 | 28885.5 |
| L 199-5 | *Theropithecus brumpti* | *Theropithecus* C | 0.897 | 0.220 | 3.616 | 36342.0 |
| L 345-3 | *Theropithecus brumpti* | *Theropithecus* C | 1.125 | 0.420 | 5.363 | 50515.1 |
| L 345-4 | *Theropithecus brumpti* | *Theropithecus* C | 0.810 | 0.261 | 1.609 | 43279.9 |
| L 440-2 | *Theropithecus brumpti* | *Theropithecus* C | 1.372 | 0.356 | 2.966 | 42413.9 |
| L 764-1 | *Theropithecus brumpti* | *Theropithecus* C | 2.097 | 0.303 | 4.911 | 41079.9 |
| L 870-1 | *Theropithecus brumpti* | *Theropithecus* C | 1.274 | 0.449 | 0.605 | 46247.0 |
| OMO 158-1973-343 | *Theropithecus brumpti* | *Theropithecus* C | 1.154 | 0.271 | 0.953 | 44151.9 |
| OMO 18-1968-370 | *Theropithecus brumpti* | *Theropithecus* C | 1.218 | 0.271 | 2.591 | 45905.7 |
| OMO 18-1969-495 | *Theropithecus brumpti* | *Theropithecus* C | 0.843 | 0.398 | 1.557 | 39026.9 |
| OMO 18-1970-1 | *Theropithecus brumpti* | *Theropithecus* C | 0.871 | 0.337 | 3.821 | 32127.4 |
| OMO 217-1973-4388 | *Theropithecus brumpti* | *Theropithecus* C | 2.352 | 0.288 | 5.936 | 56216.3 |
| L 18-23 | *Theropithecus* sp. | *Theropithecus* C | 1.295 | 0.268 | 0.128 | 45815.2 |
| L 304-13 | *Theropithecus* sp. | *Theropithecus* C | 1.012 | 0.241 | 3.815 | 43021.0 |
| L 304-15 | *Theropithecus* sp. | *Theropithecus* C | 0.792 | 0.469 | 1.254 | 43622.6 |
| L 32-270 | *Theropithecus* sp. | *Theropithecus* C | 1.093 | 0.224 | 1.068 | 28245.9 |
| L 54-30 | *Theropithecus* sp. | *Theropithecus* C | 1.909 | 0.290 | 4.309 | 51335.7 |
| L 750-10010 | *Theropithecus* sp. | *Theropithecus* C | 2.334 | 0.253 | 0.674 | 36222.2 |
| OMO 162-1973-489 | *Theropithecus* sp. | *Theropithecus* C | 1.721 | 0.401 | 0.550 | 34858.5 |
| OMO 18-1968-1053 | *Theropithecus* sp. | *Theropithecus* C | 1.311 | 0.409 | 1.575 | 37061.2 |
| OMO 18-1968-1075 | *Theropithecus* sp. | *Theropithecus* C | 1.927 | 0.493 | 0.683 | 34767.1 |
| OMO 18-1968-1105 | *Theropithecus* sp. | *Theropithecus* C | 0.963 | 0.315 | 1.297 | 37455.5 |
| OMO 18-1968-1126 | *Theropithecus* sp. | *Theropithecus* C | 0.855 | 0.273 | 1.614 | 46941.4 |
| OMO 18-1968-1407 | *Theropithecus* sp. | *Theropithecus* C | 1.622 | 0.480 | 5.068 | 49606.5 |
| OMO 18-1968-2237 | *Theropithecus* sp. | *Theropithecus* C | 1.021 | 0.593 | 3.679 | 33168.6 |
| OMO 18-1968-2249 | *Theropithecus* sp. | *Theropithecus* C | 0.859 | 0.288 | 1.704 | 43598.7 |
| OMO 18-1968-2255 | *Theropithecus* sp. | *Theropithecus* C | 1.112 | 0.558 | 7.621 | 53156.8 |
| OMO 18-1968-2260 | *Theropithecus* sp. | *Theropithecus* C | 0.809 | 0.390 | 3.227 | 28741.3 |
| OMO 18-1968-2264 | *Theropithecus* sp. | *Theropithecus* C | 1.507 | 0.502 | 4.671 | 40069.3 |
| OMO 18-1969-497 | *Theropithecus* sp. | *Theropithecus* C | 1.822 | 0.419 | 4.107 | 43600.1 |
| OMO 18-1969-510 | *Theropithecus* sp. | *Theropithecus* C | 0.700 | 0.814 | 1.888 | 13963.1 |
| OMO 18-1970-1831 | *Theropithecus* sp. | *Theropithecus* C | 1.219 | 0.311 | 3.435 | 45324.7 |
| OMO 18-1972-24 | *Theropithecus* sp. | *Theropithecus* C | 1.704 | 0.365 | 2.510 | 42488.9 |
| OMO 18-1973-2948 | *Theropithecus* sp. | *Theropithecus* C | 1.892 | 0.316 | 0.665 | 27710.7 |
| OMO 18/sup-10036 | *Theropithecus* sp. | *Theropithecus* C | 2.455 | 0.589 | 1.531 | 26546.4 |
| OMO 18/sup-10037 | *Theropithecus* sp. | *Theropithecus* C | 1.179 | 0.369 | 1.442 | 36683.3 |
| OMO 3/2-1974-336 | *Theropithecus* sp. | *Theropithecus* C | 1.377 | 0.240 | 0.510 | 43570.0 |
| OMO 3/2-1974-349 | *Theropithecus* sp. | *Theropithecus* C | 1.217 | 0.349 | 1.720 | 34448.1 |
| OMO 3/2-1974-387 | *Theropithecus* sp. | *Theropithecus* C | 1.846 | 0.452 | 3.193 | 30377.7 |
| OMO 329-10013 | *Theropithecus* sp. | *Theropithecus* C | 1.877 | 0.367 | 4.619 | 46673.3 |
| OMO 349-10017a | *Theropithecus* sp. | *Theropithecus* C | 1.366 | 0.401 | 1.284 | 25051.8 |
| OMO 349-10046 | *Theropithecus* sp. | *Theropithecus* C | 3.187 | 0.419 | 0.523 | 45659.1 |
| OMO 40-10008 | *Theropithecus* sp. | *Theropithecus* C | 0.908 | 0.316 | 4.367 | 50806.3 |
| OMO 40-1969-442 | *Theropithecus* sp. | *Theropithecus* C | 1.126 | 0.402 | 4.375 | 31290.3 |
| OMO 56-10016 | *Theropithecus* sp. | *Theropithecus* C | 2.454 | 0.631 | 1.711 | 33705.7 |
| OMO 84-1970-116 | *Theropithecus* sp. | *Theropithecus* C | 1.431 | 0.509 | 0.445 | 39039.1 |
| OMO P 791/S-1970-2984 | *Theropithecus* sp. | *Theropithecus* C | 0.818 | 0.342 | 5.881 | 54735.6 |
| L 1-334 | *Theropithecus* sp. | *Theropithecus* B | 1.432 | 0.360 | 0.187 | 30115.6 |
| L 387-10004 | *Theropithecus* sp. | *Theropithecus* B | 2.193 | 0.466 | 6.272 | 59240.0 |
| OMO 112/2-10004 | *Theropithecus* sp. | *Theropithecus* B | 4.155 | 0.360 | 0.894 | 35344.2 |
| OMO 20/4-1967-8 | *Theropithecus* sp. | *Theropithecus* B | 0.700 | 0.299 | 4.993 | 39642.6 |
| OMO 212-1973-1986 | *Theropithecus* sp. | *Theropithecus* B | 1.068 | 0.465 | 7.598 | 61911.7 |
| OMO 28-1968-1252 | *Theropithecus* sp. | *Theropithecus* B | 1.967 | 0.375 | 1.031 | 59773.2 |
| OMO 28-1968-1273 | *Theropithecus* sp. | *Theropithecus* B | 1.457 | 0.288 | 0.309 | 28459.1 |
| OMO 28-1968-1336 | *Theropithecus* sp. | *Theropithecus* B | 1.423 | 0.395 | 0.552 | 23016.6 |
| OMO 28-1968-1386 | *Theropithecus* sp. | *Theropithecus* B | 1.499 | 0.378 | 0.774 | 35355.3 |
| OMO 41-1968-1234 | *Theropithecus* sp. | *Theropithecus* B | 1.674 | 0.295 | 1.279 | 32616.1 |
| OMO 82-1970-101 | *Theropithecus* sp. | *Theropithecus* B | 1.485 | 0.787 | 1.400 | 38187.8 |

**Appendix 2:** Descriptive statistics of microwear texture parameters on both Phase I and Phase II of extinct papionins from Shungura Formation

|  | | | | *Asfc* | | |  | *HAsfc* | | |  | *epLsar* | | |  | *Tfv* | | |
| --- | --- | --- | --- | --- | --- | --- | --- | --- | --- | --- | --- | --- | --- | --- | --- | --- | --- | --- |
| Genus | Member | Facet | N | m | sd | sem |  | m | sd | sem |  | m | sd | sem |  | m | sd | sem |
| *Papio* | B | phase I | 3 | 2.762 | 3.005 | 1.735 |  | 0.523 | 0.263 | 0.152 |  | 0.994 | 0.347 | 0.201 |  | 31709.0 | 17405.4 | 10049.0 |
| *Papio* | B | phase II | 2 | 1.751 | 1.570 | 1.110 |  | 0.575 | 0.338 | 0.239 |  | 1.120 | 0.513 | 0.363 |  | 37564.2 | 27196.7 | 19231.0 |
| *Papio* | C | phase I | 7 | 1.353 | 0.936 | 0.354 |  | 0.479 | 0.134 | 0.051 |  | 2.570 | 1.108 | 0.419 |  | 38594.5 | 12851.8 | 4857.5 |
| *Papio* | C | phase II | 6 | 1.699 | 0.882 | 0.360 |  | 0.392 | 0.055 | 0.022 |  | 1.891 | 0.646 | 0.264 |  | 45301.7 | 8573.1 | 3500.0 |
| *Papio* | E | phase I | 15 | 1.804 | 0.914 | 0.236 |  | 0.436 | 0.245 | 0.063 |  | 1.853 | 1.308 | 0.338 |  | 30270.4 | 10704.0 | 2763.8 |
| *Papio* | E | phase II | 14 | 2.322 | 2.513 | 0.672 |  | 0.484 | 0.215 | 0.057 |  | 2.486 | 1.512 | 0.404 |  | 41805.1 | 11810.3 | 3156.4 |
| *Papio* | F | phase I | 12 | 1.330 | 1.000 | 0.289 |  | 0.474 | 0.145 | 0.042 |  | 2.528 | 1.816 | 0.524 |  | 27427.8 | 15663.0 | 4521.5 |
| *Papio* | F | phase II | 10 | 1.515 | 1.077 | 0.341 |  | 0.459 | 0.204 | 0.065 |  | 2.551 | 1.769 | 0.559 |  | 39147.8 | 10144.2 | 3207.9 |
| *Papio* | G | phase I | 15 | 1.803 | 0.905 | 0.234 |  | 0.547 | 0.293 | 0.076 |  | 1.531 | 0.871 | 0.225 |  | 32671.0 | 11425.6 | 2950.1 |
| *Papio* | G | phase II | 17 | 1.702 | 1.051 | 0.255 |  | 0.505 | 0.235 | 0.057 |  | 1.985 | 1.240 | 0.301 |  | 33057.5 | 15290.0 | 3708.4 |
| *Theropithecus* | B | phase I | 10 | 1.366 | 0.846 | 0.268 |  | 0.363 | 0.071 | 0.022 |  | 2.085 | 1.592 | 0.504 |  | 36958.3 | 8523.9 | 2695.5 |
| *Theropithecus* | B | phase II | 11 | 1.732 | 0.896 | 0.270 |  | 0.406 | 0.140 | 0.042 |  | 2.299 | 2.652 | 0.800 |  | 40332.9 | 13640.3 | 4112.7 |
| *Theropithecus* | C | phase I | 66 | 0.989 | 0.496 | 0.061 |  | 0.316 | 0.089 | 0.011 |  | 2.529 | 1.785 | 0.220 |  | 31544.3 | 12409.0 | 1527.4 |
| *Theropithecus* | C | phase II | 48 | 1.411 | 0.549 | 0.079 |  | 0.382 | 0.120 | 0.017 |  | 2.653 | 1.837 | 0.265 |  | 39674.8 | 8677.9 | 1252.5 |
| *Theropithecus* | D | phase I | 6 | 1.343 | 0.512 | 0.209 |  | 0.369 | 0.068 | 0.028 |  | 1.619 | 0.793 | 0.324 |  | 34300.0 | 14854.7 | 6064.4 |
| *Theropithecus* | D | phase II | 6 | 1.033 | 0.264 | 0.108 |  | 0.382 | 0.079 | 0.032 |  | 3.511 | 2.352 | 0.960 |  | 41526.0 | 5713.7 | 2332.6 |
| *Theropithecus* | E | phase I | 7 | 1.005 | 0.682 | 0.258 |  | 0.467 | 0.177 | 0.067 |  | 1.296 | 0.893 | 0.338 |  | 27412.5 | 16619.4 | 6281.5 |
| *Theropithecus* | E | phase II | 8 | 1.188 | 0.192 | 0.068 |  | 0.429 | 0.113 | 0.040 |  | 2.049 | 1.416 | 0.501 |  | 43794.6 | 8646.2 | 3056.9 |
| *Theropithecus* | F | phase I | 13 | 0.783 | 0.333 | 0.092 |  | 0.396 | 0.114 | 0.032 |  | 3.258 | 1.665 | 0.462 |  | 31433.2 | 10543.8 | 2924.3 |
| *Theropithecus* | F | phase II | 14 | 1.294 | 0.525 | 0.140 |  | 0.411 | 0.136 | 0.036 |  | 2.365 | 1.666 | 0.445 |  | 41649.0 | 11135.1 | 2976.0 |
| *Theropithecus* | G | phase I | 15 | 1.084 | 0.591 | 0.152 |  | 0.318 | 0.057 | 0.015 |  | 1.887 | 1.117 | 0.288 |  | 25719.8 | 18105.9 | 4674.9 |
| *Theropithecus* | G | phase II | 16 | 1.231 | 0.559 | 0.140 |  | 0.364 | 0.064 | 0.016 |  | 3.180 | 2.388 | 0.597 |  | 46263.2 | 8860.8 | 2215.2 |
| N: number of specimens. m: mean. sd: standard deviation. sem: standard error of the mean. | | | | | | | | | | | | | | | | | | |

**Appendix 3:** Post hoc tests of microwear texture parameters obtained on Phase II molar facets of extant and extinct Theropithecus and Papio

| HSD test on ranked *HAsfc* | | | | | | | | | | |  |  |
| --- | --- | --- | --- | --- | --- | --- | --- | --- | --- | --- | --- | --- |
|  | Genus | Sample | 1 | 2 | 3 | 4 | 5 | 6 | 7 | 8 | 9 | 10 |
| 1 | *Papio* | E |  |  |  |  |  |  |  |  |  |  |
| 2 | *Papio* | F | 1.000 |  |  |  |  |  |  |  |  |  |
| 3 | *Papio* | G | 1.000 | 1.000 |  |  |  |  |  |  |  |  |
| 4 | *Papio* | *P. h. a.* | 0.398 | 0.259 | 0.476 |  |  |  |  |  |  |  |
| 5 | *Papio* | *P. h. c.* | 0.580 | 0.399 | 0.671 | 1.000 |  |  |  |  |  |  |
| 6 | *Papio* | *P. h. h.* | 0.441 | 0.299 | 0.516 | 1.000 | 0.999 |  |  |  |  |  |
| 7 | *Theropithecus* | E | 1.000 | 1.000 | 1.000 | 0.401 | 0.562 | 0.400 |  |  |  |  |
| 8 | *Theropithecus* | F | 0.986 | 1.000 | 0.939 | **0.030** | 0.058 | 0.076 | 1.000 |  |  |  |
| 9 | *Theropithecus* | G | 0.522 | 0.903 | 0.304 | **0.001** | **0.001** | **0.006** | 0.913 | 0.992 |  |  |
| 10 | *Theropithecus* | *T.g.* | 0.987 | 1.000 | 0.934 | **0.017** | **0.033** | 0.063 | 1.000 | 1.000 | 0.971 |  |
| Bold p-values are significant. | | | | | | | | | | | | |

| LSD test on ranked *HAsfc* | | | | | | | | | | |  |  |
| --- | --- | --- | --- | --- | --- | --- | --- | --- | --- | --- | --- | --- |
|  | Genus | Sample | 1 | 2 | 3 | 4 | 5 | 6 | 7 | 8 | 9 | 10 |
| 1 | *Papio* | E |  |  |  |  |  |  |  |  |  |  |
| 2 | *Papio* | F | 0.660 |  |  |  |  |  |  |  |  |  |
| 3 | *Papio* | G | 0.822 | 0.509 |  |  |  |  |  |  |  |  |
| 4 | *Papio* | *P. h. a.* | **0.023** | **0.012** | **0.031** |  |  |  |  |  |  |  |
| 5 | *Papio* | *P. h. c.* | **0.045** | **0.023** | 0.060 | 0.710 |  |  |  |  |  |  |
| 6 | *Papio* | *P. h. h.* | **0.027** | **0.015** | **0.036** | 0.627 | 0.439 |  |  |  |  |  |
| 7 | *Theropithecus* | E | 0.735 | 0.946 | 0.590 | **0.024** | **0.042** | **0.023** |  |  |  |  |
| 8 | *Theropithecus* | F | 0.284 | 0.589 | 0.179 | **0.001** | **0.002** | **0.003** | 0.564 |  |  |  |
| 9 | *Theropithecus* | G | **0.037** | 0.145 | **0.016** | **< 0.001** | **< 0.001** | **< 0.001** | 0.153 | 0.319 |  |  |
| 10 | *Theropithecus* | *T.g.* | 0.289 | 0.628 | 0.173 | **0.001** | **0.001** | **0.002** | 0.599 | 0.919 | 0.233 |  |
| Bold p-values are significant. | | | | | | | | | | | | |

| HSD test on ranked *epLsar* | | | | | | | | | | |  |  |
| --- | --- | --- | --- | --- | --- | --- | --- | --- | --- | --- | --- | --- |
|  | Genus | Sample | 1 | 2 | 3 | 4 | 5 | 6 | 7 | 8 | 9 | 10 |
| 1 | *Papio* | E |  |  |  |  |  |  |  |  |  |  |
| 2 | *Papio* | F | 1.000 |  |  |  |  |  |  |  |  |  |
| 3 | *Papio* | G | 0.997 | 0.999 |  |  |  |  |  |  |  |  |
| 4 | *Papio* | *P. h. a.* | 0.938 | 0.962 | 0.377 |  |  |  |  |  |  |  |
| 5 | *Papio* | *P. h. c.* | 1.000 | 1.000 | 0.804 | 0.999 |  |  |  |  |  |  |
| 6 | *Papio* | *P. h. h.* | 1.000 | 1.000 | 0.957 | 1.000 | 1.000 |  |  |  |  |  |
| 7 | *Theropithecus* | E | 0.999 | 1.000 | 1.000 | 0.680 | 0.943 | 0.981 |  |  |  |  |
| 8 | *Theropithecus* | F | 1.000 | 1.000 | 1.000 | 0.800 | 0.989 | 0.998 | 1.000 |  |  |  |
| 9 | *Theropithecus* | G | 1.000 | 1.000 | 0.864 | 0.999 | 1.000 | 1.000 | 0.962 | 0.995 |  |  |
| 10 | *Theropithecus* | *T.g.* | 0.351 | 0.494 | **0.023** | 0.995 | 0.744 | 0.977 | 0.168 | 0.174 | 0.732 |  |
| Bold p-values are significant. | | | | | | | | | | | | |

| LSD test on ranked *epLsar* | | | | | | | | | | |  |  |
| --- | --- | --- | --- | --- | --- | --- | --- | --- | --- | --- | --- | --- |
|  | Genus | Sample | 1 | 2 | 3 | 4 | 5 | 6 | 7 | 8 | 9 | 10 |
| 1 | *Papio* | E |  |  |  |  |  |  |  |  |  |  |
| 2 | *Papio* | F | 0.986 |  |  |  |  |  |  |  |  |  |
| 3 | *Papio* | G | 0.375 | 0.432 |  |  |  |  |  |  |  |  |
| 4 | *Papio* | *P. h. a.* | 0.178 | 0.213 | **0.021** |  |  |  |  |  |  |  |
| 5 | *Papio* | *P. h. c.* | 0.490 | 0.520 | 0.095 | 0.464 |  |  |  |  |  |  |
| 6 | *Papio* | *P. h. h.* | 0.559 | 0.571 | 0.203 | 0.652 | 0.935 |  |  |  |  |  |
| 7 | *Theropithecus* | E | 0.470 | 0.510 | 0.999 | 0.062 | 0.184 | 0.263 |  |  |  |  |
| 8 | *Theropithecus* | F | 0.744 | 0.779 | 0.585 | 0.094 | 0.300 | 0.403 | 0.657 |  |  |  |
| 9 | *Theropithecus* | G | 0.545 | 0.570 | 0.121 | 0.435 | 0.943 | 0.895 | 0.212 | 0.346 |  |  |
| 10 | *Theropithecus* | *T.g.* | **0.019** | **0.033** | **0.001** | 0.346 | 0.077 | 0.247 | **0.007** | **0.007** | 0.074 |  |
| Bold p-values are significant. | | | | | | | | | | | | |

**Appendix 4:** Descriptive statistics of microwear texture parameters on both Phase I and Phase II facets of *T. brumpti* and *T. oswaldi*

|  |  |  |  | *Asfc* | | |  | *HAsfc* | | |  | *epLsar* | | |  | *Tfv* | | |
| --- | --- | --- | --- | --- | --- | --- | --- | --- | --- | --- | --- | --- | --- | --- | --- | --- | --- | --- |
| Species | Facet | N |  | m | sd | sem |  | m | sd | sem |  | m | sd | sem |  | m | sd | sem |
| *Theropithecus brumpti* | Phase I | 32 |  | 1.164 | 0.507 | 0.090 |  | 0.320 | 0.104 | 0.018 |  | 2.423 | 1.266 | 0.224 |  | 28614.6 | 12768.0 | 2257.1 |
| *Theropithecus brumpti* | Phase II | 24 |  | 1.254 | 0.449 | 0.092 |  | 0.352 | 0.077 | 0.016 |  | 2.936 | 1.647 | 0.336 |  | 42902.0 | 7168.6 | 1463.3 |
| *Theropithecus oswaldi* | Phase I | 6 |  | 0.997 | 0.274 | 0.112 |  | 0.306 | 0.109 | 0.044 |  | 1.898 | 0.889 | 0.363 |  | 24634.2 | 12582.3 | 5136.7 |
| *Theropithecus oswaldi* | Phase II | 3 |  | 1.262 | 0.295 | 0.170 |  | 0.346 | 0.013 | 0.008 |  | 2.454 | 0.664 | 0.383 |  | 46815.7 | 5699.3 | 3290.5 |
| N: number of specimens. m: mean. sd: standard deviation. sem: standard error of the mean. | | | | | | | | | | | | | | | | | | |

**Appendix 5:** Univariate Analyses of Variance of each texture parameter on Phase I and Phase II molar facets of Theropithecus brumpti and T. oswaldi

| ANOVAs | Variable | F | df | p |
| --- | --- | --- | --- | --- |
| ***Phase I facets*** |  |  |  |  |
|  |  |  |  |  |
|  | *Asfc* | 0.354 | 1; 36 | 0.555 |
|  | *HAsfc* | 0.100 | 1; 36 | 0.754 |
|  | *epLsar* | 0.921 | 1; 36 | 0.344 |
|  | *Tfv* | 0.354 | 1; 36 | 0.555 |
|  |  |  |  |  |
| ***Phase II facets*** |  |  |  |  |
|  |  |  |  |  |
|  | *Asfc* | 0.284 | 1; 25 | 0.599 |
|  | *HAsfc* | 0.023 | 1; 25 | 0.881 |
|  | *epLsar* | 0 | 1; 25 | 1 |
|  | *Tfv* | 1.358 | 1; 25 | 0.255 |
| F: value of the test. df: degrees of freedom. p: p-value. Bold p-values are significant. | | | | |

**Appendix 6:** Weight of each principal component and contribution of each variable to the axes for the four PCAs performed on Phase I and Phase II molar facets of extinct Theropithecus and Papio

| Component | eigenvalues | % variance | cumulative eigenvalues | cumulative % variance |
| --- | --- | --- | --- | --- |
| Phase I facets on extinct *Theropithecus* |  |  |  |  |
|  |  |  |  |  |
| PC1 | 1.327 | 33.17 | 1.327 | 33.17 |
| PC2 | 1.249 | 31.23 | 2.576 | 64.40 |
| PC3 | 0.789 | 19.73 | 3.365 | 84.13 |
| PC4 | 0.635 | 15.87 | 4 | 100 |
|  |  |  |  |  |
| Phase I facets on extinct *Papio* |  |  |  |  |
|  |  |  |  |  |
| PC1 | 1.457 | 36.42 | 1.457 | 36.42 |
| PC2 | 1.164 | 29.11 | 2.621 | 65.53 |
| PC3 | 0.940 | 23.49 | 3.561 | 89.02 |
| PC4 | 0.439 | 10.98 | 4 | 100 |
|  |  |  |  |  |
| Phase II facets on extinct *Theropithecus* |  |  |  |  |
|  |  |  |  |  |
| PC1 | 1.495 | 37.38 | 1.495 | 37.38 |
| PC2 | 1.030 | 25.76 | 2.525 | 63.14 |
| PC3 | 0.987 | 24.67 | 3.512 | 87.81 |
| PC4 | 0.487 | 12.18 | 4 | 100 |
|  |  |  |  |  |
| Phase II facets on extinct *Papio* |  |  |  |  |
|  |  |  |  |  |
| PC1 | 1.605 | 40.12 | 1.605 | 40.12 |
| PC2 | 1.043 | 26.08 | 2.648 | 66.2 |
| PC3 | 0.850 | 21.26 | 3.498 | 87.46 |
| PC4 | 0.501 | 12.53 | 4 | 100 |

|  | PC1 | |  | PC2 | |  | PC3 | |  | PC4 | |
| --- | --- | --- | --- | --- | --- | --- | --- | --- | --- | --- | --- |
|  | r | r2 |  | r | r2 |  | r | r2 |  | r | r2 |
| Phase I *Papio* |  |  |  |  |  |  |  |  |  |  |  |
|  |  |  |  |  |  |  |  |  |  |  |  |
| *Asfc* | -0.738 | 0.545 |  | -0.070 | 0.005 |  | 0.078 | 0.006 |  | 0.667 | 0.444 |
| *HAsfc* | -0.284 | 0.081 |  | 0.386 | 0.149 |  | -0.860 | 0.740 |  | -0.173 | 0.030 |
| *epLsar* | 0.436 | 0.190 |  | 0.710 | 0.504 |  | 0.064 | 0.004 |  | 0.550 | 0.302 |
| *Tfv* | -0.430 | 0.185 |  | 0.585 | 0.342 |  | 0.500 | 0.250 |  | -0.473 | 0.224 |
|  |  |  |  |  |  |  |  |  |  |  |  |
| Phase II *Papio* |  |  |  |  |  |  |  |  |  |  |  |
|  |  |  |  |  |  |  |  |  |  |  |  |
| *Asfc* | -0.666 | 0.443 |  | 0.115 | 0.013 |  | -0.085 | 0.007 |  | 0.732 | 0.536 |
| *HAsfc* | -0.596 | 0.355 |  | -0.104 | 0.011 |  | -0.537 | 0.289 |  | -0.588 | 0.345 |
| *epLsar* | 0.198 | 0.039 |  | -0.872 | 0.760 |  | -0.353 | 0.124 |  | 0.276 | 0.076 |
| *Tfv* | -0.403 | 0.163 |  | -0.464 | 0.216 |  | 0.761 | 0.580 |  | -0.205 | 0.042 |
|  |  |  |  |  |  |  |  |  |  |  |  |
| Phase I *Theropithecus* |  |  |  |  |  |  |  |  |  |  |  |
|  |  |  |  |  |  |  |  |  |  |  |  |
| *Asfc* | 0.674 | 0.455 |  | 0.169 | 0.028 |  | 0.463 | 0.215 |  | -0.550 | 0.302 |
| *epLsar* | -0.415 | 0.172 |  | 0.612 | 0.375 |  | -0.321 | 0.103 |  | -0.592 | 0.350 |
| *HAsfc* | 0.610 | 0.372 |  | 0.183 | 0.033 |  | -0.752 | 0.566 |  | 0.170 | 0.029 |
| *Tfv* | 0.038 | 0.001 |  | 0.751 | 0.563 |  | 0.341 | 0.116 |  | 0.565 | 0.319 |
|  |  |  |  |  |  |  |  |  |  |  |  |
| Phase II *Theropithecus* |  |  |  |  |  |  |  |  |  |  |  |
|  |  |  |  |  |  |  |  |  |  |  |  |
| *Asfc* | 0.253 | 0.064 |  | 0.660 | 0.435 |  | 0.651 | 0.423 |  | -0.278 | 0.077 |
| *epLsar* | -0.677 | 0.458 |  | -0.230 | 0.053 |  | 0.212 | 0.045 |  | -0.667 | 0.444 |
| *HAsfc* | 0.207 | 0.043 |  | -0.657 | 0.432 |  | 0.686 | 0.470 |  | 0.234 | 0.055 |
| *Tfv* | -0.660 | 0.435 |  | 0.283 | 0.080 |  | 0.247 | 0.061 |  | 0.651 | 0.424 |
| r: correlation coefficients between the variables and the principal components. r2: proportion of the variance of the principal component explained by the considered variable. | | | | | | | | | | | |

**Appendix 7:** ANOVAs ran on the four principal components of each PCA performed on Phase I and Phase II molar facets of extinct Theropithecus and Papio

| ANOVAs | Variable | F | df | p |
| --- | --- | --- | --- | --- |
| ***Phase I facets*** |  |  |  |  |
|  |  |  |  |  |
| *Theropithecus* | PC1 | 3.974 | 5, 111 | **0.002** |
|  | PC2 | 1.426 | 5, 111 | 0.220 |
|  | PC3 | 3.901 | 5, 111 | **0.003** |
|  | PC4 | 0.542 | 5, 111 | 0.744 |
|  |  |  |  |  |
| *Papio* | PC1 | 1.521 | 5, 47 | 0.201 |
|  | PC2 | 1.322 | 5, 47 | 0.271 |
|  | PC3 | 0.522 | 5, 47 | 0.758 |
|  | PC4 | 0.875 | 5, 47 | 0.505 |
|  |  |  |  |  |
| ***Phase II facets*** |  |  |  |  |
|  |  |  |  |  |
| *Theropithecus* | PC1 | 1.183 | 5, 97 | 0.323 |
|  | PC2 | 0.847 | 5, 97 | 0.520 |
|  | PC3 | 0.249 | 5, 97 | 0.939 |
|  | PC4 | 1.433 | 5, 97 | 0.219 |
|  |  |  |  |  |
| *Papio* | PC1 | 0.191 | 4, 44 | 0.942 |
|  | PC2 | 1.047 | 4, 44 | 0.394 |
|  | PC3 | 1.746 | 4, 44 | 0.157 |
|  | PC4 | 0.664 | 4, 44 | 0.621 |
| F: value of the test. df: degrees of freedom. p: p-value. Bold p-values are significant. | | | | |

**Appendix 8:** Between-members pairwise comparisons of PC1 coordinates on Phase I molar facets of Theropithecus

| Members | | Tukey’s HSD | Fisher’s LSD |
| --- | --- | --- | --- |
| vs | | p | p |
| B | C | 0.088 | **0.009** |
| B | D | 0.988 | 0.523 |
| B | E | 0.998 | 0.678 |
| B | F | 0.411 | 0.061 |
| B | lower G | 0.723 | 0.162 |
| C | D | **0.049** | **0.004** |
| C | E | 0.065 | **0.006** |
| C | F | 0.999 | 0.715 |
| C | lower G | 0.851 | 0.246 |
| D | E | 1 | 0.821 |
| D | F | 0.209 | **0.024** |
| D | lower G | 0.424 | 0.064 |
| E | F | 0.276 | **0.035** |
| E | lower G | 0.534 | 0.092 |
| F | lower G | 0.992 | 0.558 |
| p: p-value. Bold p-values are significant. | | | |

**Appendix 9:** Between-members pairwise comparisons of PC3 coordinates on Phase I molar facets of Theropithecus

| Members | | Tukey’s HSD | Fisher’s LSD |
| --- | --- | --- | --- |
| vs | | p | p |
| B | C | 0.967 | 0.427 |
| B | D | 1.000 | 0.888 |
| B | E | 0.130 | **0.014** |
| B | F | **0.027** | **0.002** |
| B | lower G | 0.992 | 0.565 |
| C | D | 0.997 | 0.644 |
| C | E | 0.154 | **0.017** |
| C | F | **0.010** | **0.001** |
| C | lower G | 1.000 | 0.903 |
| D | E | 0.299 | **0.039** |
| D | F | 0.129 | **0.013** |
| D | lower G | 0.999 | 0.737 |
| E | F | 1.000 | 0.872 |
| E | lower G | 0.251 | **0.031** |
| F | lower G | 0.058 | **0.005** |
| p: p-value. Bold p-values are significant. | | | |


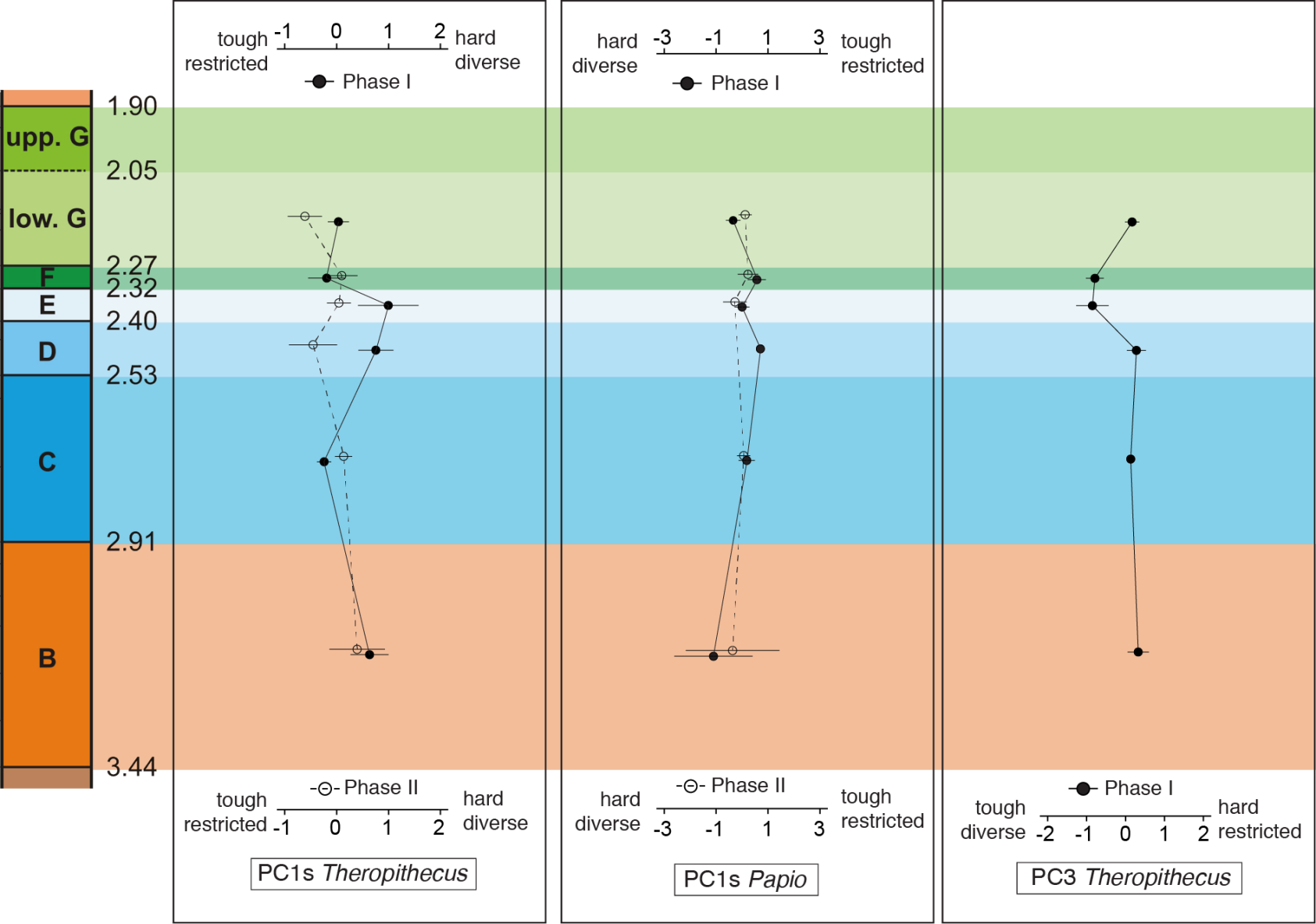
**Appendix 10:** Temporal fluctuations in dental microwear textures on Phase I and Phase II molar facets of extinct Theropithecus and Papio from the Shungura Formation. Only PC1 and PC3 on Phase I molar facets of Theropithecus show significant differences between geological members. Symbols on the curves represent means and error bars represent standard errors of means.
